# Septate junction proteins are required for cell shape changes, actomyosin reorganization and cell adhesion during dorsal closure in *Drosophila*

**DOI:** 10.3389/fcell.2022.947444

**Published:** 2022-09-27

**Authors:** Oindrila De, Clinton Rice, Teresa Zulueta-Coarasa, Rodrigo Fernandez-Gonzalez, Robert E. Ward

**Affiliations:** ^1^ Department of Biology, Case Western Reserve University, Cleveland, Ohio, United States; ^2^ Department of Molecular Biosciences, University of Kansas, Lawrence, Kansas, United States; ^3^ Institute of Biomedical Engineering, University of Toronto, Toronto, ON, Canada

**Keywords:** septate junction, dorsal closure, adhesion, cell shape change, actomyosin

## Abstract

Septate junctions (SJs) serve as occluding barriers in invertebrate epithelia. In *Drosophila*, at least 30 genes are required for the formation or maintenance of SJs. Interestingly, loss-of-function mutations in core SJ components are embryonic lethal, with defects in developmental events such as head involution and dorsal closure (DC) that occur prior to the formation of a mature SJ, indicating a role for these proteins in mid-embryogenesis independent of their occluding function. To understand this novel function in development, we examined loss-of-function mutations in three core SJ proteins during the process of DC. DC occurs during mid-embryogenesis to seal a dorsal gap in the epidermis following germ band retraction. Closure is driven by contraction of the extraembryonic amnioserosa cells that temporarily cover the dorsal surface and by cell shape changes (elongation) of lateral epidermal cells that bring the contralateral sheets together at the dorsal midline. Using live imaging and examination of fixed tissues, we show that early events in DC occur normally in SJ mutant embryos, but during later closure, *coracle*, *Macroglobulin complement-related* and *Neurexin-IV* mutant embryos exhibit slower rates of closure and display aberrant cells shapes in the dorsolateral epidermis, including dorsoventral length and apical surface area. SJ mutant embryos also show mild defects in actomyosin structures along the leading edge, but laser cutting experiments suggest similar tension and viscoelastic properties in SJ mutant versus wild type epidermis. In a high percentage of SJ mutant embryos, the epidermis tears free from the amnioserosa near the end of DC and live imaging and immunostaining reveal reduced levels of E-cadherin, suggesting that defective adhesion may be responsible for these tears. Supporting this notion, reducing E-cadherin by half significantly enhances the penetrance of DC defects in *coracle* mutant embryos.

## Introduction

Morphogenesis involves highly coordinated cell shape changes and cell rearrangements, which require precise regulation of cytoskeletal reorganization and cell adhesion. In general, intercellular signaling events mediate the orchestrated movement of cells by regulating actomyosin dynamics, while cell adhesive complexes maintain tissue integrity. However, the cellular mechanisms that ensure epithelial integrity during dynamic changes in a tissue undergoing morphogenesis remain unclear. To expand our understanding of epithelial morphogenesis and gain critical insights into the interplay between cytoskeletal dynamics and adhesion, it is important to identify new genes and uncover novel mechanisms. Previously, we isolated an allele of *Macroglobulin-complement related* (*Mcr*) from an EMS mutagenesis screen to identify genes required for *Drosophila* imaginal disc morphogenesis (Ward et al., 2003). We went on to show that loss-of-function mutations in *Mcr* resulted in embryonic lethality, characterized by defects in the processes of dorsal closure, head involution, and tracheal and salivary gland organogenesis (Hall et al., 2014). Interestingly, Mcr localizes to the septate junction (SJ) and serves as a core component required for the organization and function of the junction (Hall et al., 2014), raising the possibility that SJs or specific SJ proteins may have a role in morphogenesis (reviewed in Rice et al., 2021).

The SJ consists of a large group of interdependent proteins that provide an occluding barrier to the invertebrate epithelium, similar to the function of tight junctions in vertebrates (Lord and DiBona, 1976; Ward et al., 1998; Genova and Fehon, 2003). In *Drosophila*, more than 30 proteins have been identified as required for the establishment or maintenance of SJs (Rice et al., 2021). The “core” SJ complex includes proteins from the claudin family (e.g., Megatrachea, Sinuous, Kune-Kune) (Behr et al., 2003; Wu et al., 2004; Nelson et al., 2010), cell-adhesion molecules (e.g., Neurexin-IV, Neuroglian, Gliotactin, etc.) (Baumgartner et al., 1996; Genova and Fehon, 2003), and cytoplasmic proteins (e.g,. Coracle, Varicose) (Fehon et al., 1994; Wu et al., 2007) (the molecular organization of representative core SJ proteins is depicted in Supplementary Figure S1). In addition, members of the leukocyte antigen 6 (Ly6) family of proteins (e.g. Boudin, Crooked, Coiled) and proteins involved in membrane vesicle trafficking (e.g., Rab5, Rab11, Dynamin) are required for SJ maturation (Hijazi et al., 2009; Nilton et al., 2010; Tiklová et al., 2010). Core components of the junction initially localize along the lateral membrane beginning from stage 12 of embryogenesis. During stages 14–15 of embryogenesis, these proteins are endocytosed and recycled back to the membrane, where they are enriched at an apical lateral region just basal to the adherens junction (Tiklová et al., 2010). The SJ is physiologically “tight” by the end of stage 15 and has a mature appearance of ladder-like septa (via ultrastructural analysis) by stage 17 (Tepass and Hartenstein, 1994; Paul et al., 2003).

To investigate developmental requirements for core SJ proteins during embryogenesis, we previously examined loss-of-function mutations in a large subset of core SJ genes and found that they were all embryonic lethal and showed defects in head involution (Hall and Ward, 2016). Several of these mutations, including *Mcr*, *coracle* (*cora*), and *Neurexin-IV* (*Nrx-IV*), also showed substantial defects in dorsal closure (DC). *Mcr* encodes a transmembrane protein with an α-2-macroglobulin domain and belongs to the family of thioester-containing proteins (Hall et al., 2014). *cora* enocodes a cytoplasmic protein with an N-terminal FERM (Protein 4.1/Ezrin/Radixin/Moesin) domain (Fehon et al., 1994). *Nrx-IV* encodes a transmembrane protein with a large extracellular domain consisting of laminin G and epidermal growth factor (EGF)-like motifs (Baumgartner et al., 1996). Interestingly, head involution and DC occur during stages 13–15 of embryogenesis, prior to the formation of a mature SJ (and its diffusion barrier), suggesting a non-occluding function of SJ proteins in morphogenesis. However, the specific nature of the requirement of SJ proteins in these developmental processes and the underlying cellular mechanisms are unknown.

DC in *Drosophila* serves as a well-established model to study cellular mechanisms of morphogenesis. After the germ band retracts during mid-embryogenesis, the dorsal surface of the embryo consists of an extraembryonic tissue known as the amnioserosa. DC describes the processes that stretch the lateral epidermal sheets over the dorsal surface to enclose the embryo. These processes include actomyosin-dependent pulsatile contractions of amnioserosa cells that generate the major pulling force on the lateral epidermis (Kiehart et al., 2000; Solon et al., 2009). A subset of these amnioserosa cells delaminate during closure (Toyama et al., 2008; Sokolow et al., 2012), further reducing the surface area of this tissue. The main signaling event initiating closure is the activation of Jun N-terminal kinase (JNK) pathway in the dorsal-most row of epidermal (DME) cells that line the interface (referred to as the “leading edge”) between the amnioserosa and the lateral epidermis. JNK activation at the leading edge leads to expression of Decapentaplegic (Dpp) in the DME cells that promotes the formation of a contractile supracellular actomyosin cable at the leading edge (Hou et al., 1997; Zahedi et al., 2008). Although the cable is dispensable for closure, it contributes to the smoothness of the leading edge and epithelial continuity as the two flanking lateral epidermal sheets fuse (Ducuing and Vincent, 2016; Pasakarnis et al., 2016). As DC initiates, the DME cells elongate along the dorsoventral axis, which is followed by dorsoventral elongation of the adjacent rows of cells (Kiehart et al., 2000). Near the end of DC, DME cells extend dynamic F-actin-rich filopodia that interdigitate to promote correct cell-cell adhesion, match compartment boundaries from the contralateral tissues, and form a seamless epithelium (Jacinto et al., 2000; Millard and Martin, 2008).

Multiple components of cellular junctions are required for effective DC. The adherens junction serves a central role as both an adhesive contact between cells and connection to the actin cytoskeleton. The central complex consists of the adhesion protein E-cadherin and the cytoplasmic proteins catenin (Armadillo in *Drosophila*) and catenin. E-cadherin and Armadillo display dynamic patterns of expression during DC (Gorfinkiel and Arias, 2007). Early in DC, both proteins are strongly expressed at the leading edge, whereas later in the process, E-cadherin and Armadillo accumulate in puncta that serve as actin nucleating centers along the leading edge, but are not strongly expressed along the entire interface. During the zippering phase of closure, a continuous line of expression forms as the contralateral cells meet at the dorsal midline. Although both proteins are expressed maternally, functional studies using zygotic loss of function alleles reveal essential roles for E-cadherin and Armadillo in maintaining adhesion between the amnioserosa and the DME cells, with frequent tears occurring at the leading edge late in DC in mutants (Gorfinkiel and Arias, 2007). Proteins that interact with these adherens junction proteins are also critically required for DC. Included in this group are Ajuba (Razzell et al., 2018), which regulates adhesion in the adherens junction in response to tension, and Canoe (the *Drosophila* homolog of Afadin) and Polychaetoid (the *Drosophila* homolog of ZO-1) that collaborate to regulate junctional-cytoskeletal interactions (Manning et al., 2019). SJ proteins also show dynamic patterns of expression during DC, in which these proteins are not expressed (or are expressed at very low levels) in the amnioserosa and are excluded from the leading edge in DME cells, but are expressed in the lateral membrane at other cell-cell contacts in the epidermis (Gorfinkiel and Arias, 2007). SJ proteins are gradually enriched at the interphase of contralateral DME cells as they fuse during the zippering phase of DC. As described above, several SJ proteins are required for DC, although their cellular functions in this process have not been described.

In the present study, we set out to explore the role of core SJ proteins during DC. Using mutations in *Mcr*, *Nrx-IV* and *cora*, we performed fixed tissue and live imagining analyses to examine cellular defects in mutant embryos during DC. We show that initiation of DC occurs normally in SJ mutant embryos. As DC progresses, however, SJ mutant embryos exhibit slower rates of closure or arrest prior to the completion of the process. At later stages of DC in SJ mutant embryos, defects in epithelial cell shape and actomyosin structures become progressively aberrant. In addition, the epidermis tears away from the amnioserosa near the completion of DC in many SJ mutant embryos. Further analysis demonstrates that these defects are likely due to a loss in adhesion rather than increased tension, suggesting a role for SJ proteins in the maintenance of adhesive structures during tissue morphogenesis.

## Results

### Live imaging of SJ mutant embryos reveals defects in late stages of DC

We previously determined that several SJ genes are required for morphogenetic events during mid-embryogenesis, including DC, head involution and salivary gland morphogenesis (Hall and Ward, 2016). Defects in head involution and DC were determined by terminal phenotypes using cuticle preparations, which likely underestimated their penetrance (see for example Mortensen et al., 2018; Fogerson et al., 2020), and also failed to reveal how these defects occur. We therefore employed a live imaging approach using strong loss of function alleles of the three genes that gave the strongest DC defects: *Nrx-IV* ^*4304*^, *cora* ^*4*^, and *Mcr* ^*1*^. Mutants for each of these genes exhibit a high frequency of dorsal cuticle defects, ranging from small circular scabs to large holes encompassing most of the dorsal surface. These phenotypes were more penetrant and generally more severe in *cora* ^*4*^ and *Nrx-IV* ^*4304*^ mutants than in *Mcr* ^*1*^ mutants (Hall and Ward, 2016).

**FIGURE 1:**
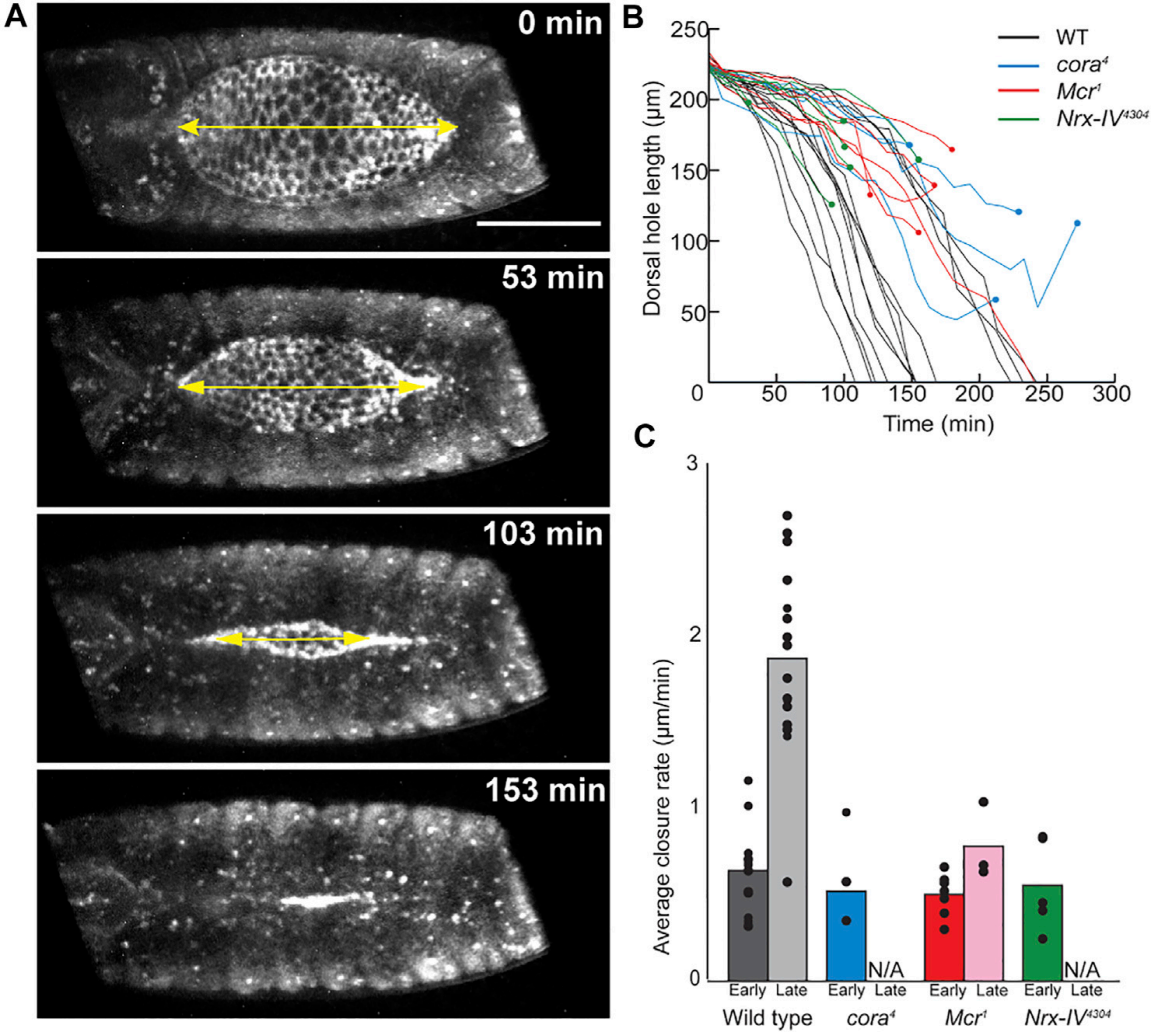
Live image analysis of DC in wild type and SJ mutant embryos. **(A)** Example of still images from time lapse series of wild type embryo, showing a line segment (yellow double headed arrow) used to measure the length across the anterior-posterior axis of the dorsal hole. **(B)** Change in length of dorsal hole over time. Circles represent timepoints when tearing occurred. **(C)** Average rate of dorsal hole shortening during early (length > 190 μm) and late (length < 190 μm) closure. Only late closure data from embryos that closed without puckering is included. Scale bar = 100 µm.

To determine how DC defects arose in these mutants, we performed live imaging by crossing a tdTomato-tagged knock-in *shotgun* (*shg*) allele (Huang et al., 2009) into the SJ mutant lines. We dechorionated and mounted the embryos just after they completed germ band retraction for this analysis. The embryos were imaged from the dorsal side to allow us to measure the length of the open dorsal surface from canthus to canthus along the anterior-posterior axis (Figure 1A, Supplementary Movies S1-4). We synchronized the timing of the data analysis by beginning closure time (*t* = 0) when the dorsal hole had a length between 220 and 230 μm and imaged them until they completed closure, ceased closing or we observed a tear between the epidermis and amnioserosa (see below). In wild type embryos, we observed a bimodal rate of closure. The initial rate of closure (shortening along the A/P length of open hole) was approximately 0.64 μm/min (*n* = 12) and lasted until the hole reached approximately 190 μm in length (Figures 1A‐C). At this inflection point, the rate of closure increased to 1.88 μm/min until closure was complete (Figures 1B,C). SJ mutant embryos proceeded through early dorsal closure at a rate that was not significantly different from wild type embryos, closing at an average rate of 0.52 μm/min (*n* = 5) for *cora* ^*4*^ embryos, 0.50 μm/min (*n* = 7) for *Mcr* ^*1*^ embryos, and 0.57 μm/min (*n* = 5) for *Nrx-IV* ^*4304*^ embryos (Figures 1B,C). During late closure, however, SJ mutant embryos generally failed to enter the fast phase of closure and either continued towards closure at a slow rate or arrested prior to closure (Figure 1B).

The observation that SJ mutant embryos showed an initial rate of closure similar to that of wild type embryos suggested that initiation and early phases of DC may be unaffected in these embryos. To confirm this notion, we examined the activation of the JNK pathway in SJ mutant embryos. In wild type embryos, the initiation of closure requires the activation of the JNK pathway in the DME cells, resulting in expression of *decapentaplegic* (*dpp*) and *puckered* (*puc*) in these cells (Riesgo-Escovar and Hafen 1997). We therefore crossed an enhancer trap allele of *puc* (*puc* ^*E69*^) onto the *Nrx-IV* ^*4304*^ chromosomes and examined *puc* expression in homozygous and heterozygous embryos as a readout of JNK signaling. These embryos were stained with an antibody against E-cadherin to outline cells and β-gal to label *puc* expression. During initiation of DC (stage 13), all heterozygous embryos (*n* ≥ 9 for each genotype) displayed a single row of *puc* expressing DME cells, marking the activation of JNK signaling pathway (Supplementary Figure S2). Similarly, *puc* is expressed in the DME cells of all homozygous mutant stage 13 embryos (Supplementary Figure S2), indicating that JNK activation during initiation of closure is unaffected in these animals. To determine if JNK activation is normal in other SJ mutations, we performed similar experiments with two other third chromosome SJ genes, *Contactin* (*Cont* ^*ex956*^) and *Transferrin 2* (*Tsf2* ^*KG01571*^) and found that JNK activation was unaffected in these mutants as well (Supplementary Figure S2).

### Cell shape and actomyosin defects arise during later stages of closure

**FIGURE 2:**
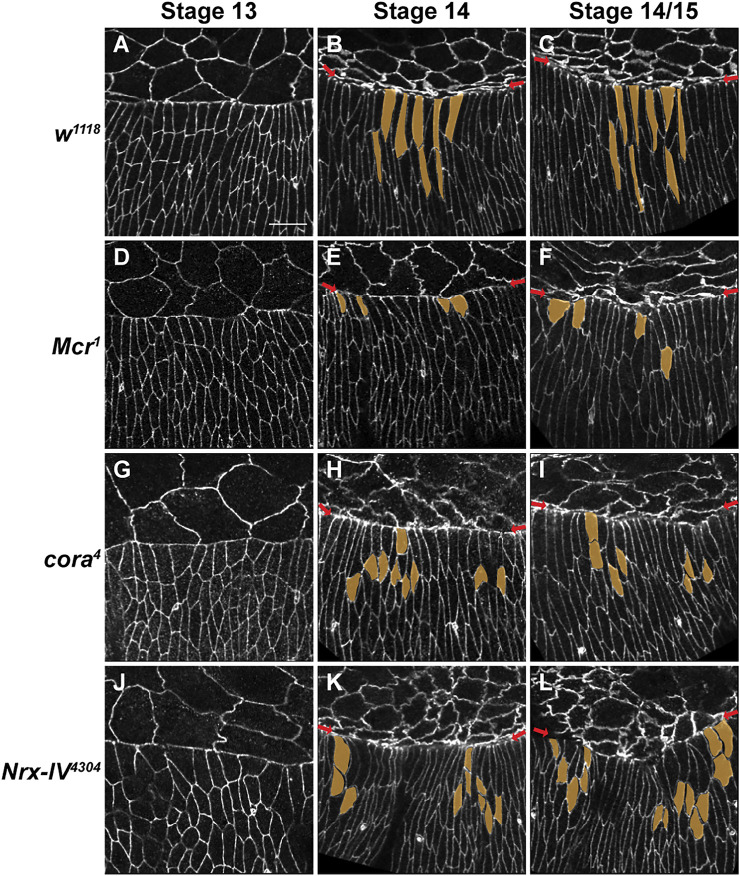
SJ mutants have defects in epidermal cell shape and organization of the leading-edge during later stages in closure. **(A–L)** Confocal optical sections of stage 13, 14 and 14-15 wild type, *Mcr*, *cora* ^*4*^ and *Nrx-IV* ^*4304*^ embryos stained with an antibody against E-cadherin. (**A–C)** During closure in wild type embryos, DME cells along the leading edge (red arrows) smoothen into a neatly organized row of cells that is elongated along the dorsoventral axis. The row of cells immediately ventral to the DME also elongate dorsoventrally. **(D,G,J)** DME cells in stage 13 SJ mutant embryos elongate along the dorsoventral axis and show only mild defects in the lateral epidermis. **(E,H,K)** In stage 14 SJ mutant embryos, groups of lateral epidermal cells fail to elongate to the same extent as wild type (segmented in yellow). **(F,I,L)** The leading edge in stage 14-15 SJ mutant embryos is irregular (red arrows). Groups of cells at the leading edge and following row exhibit defects in elongation. These cells appear more constricted or wider (segmented in yellow). Representative DME and second row cells are pseudocolored in yellow. Scale bar = 10 µm.

We next examined cell shape changes in the DME and lateral epidermal cells in wild type and SJ mutant embryos. To examine cell shapes, we fixed wild type *w* ^*1118*^, *Mcr* ^*1*^, *cora* ^*4*^, and *Nrx-IV* ^*4304*^ embryos and stained them with antibodies against E-cadherin. In early stage 13 *w* ^*1118*^ embryos, the initial phase of DC is marked by the scalloped appearance of the epidermal leading edge due to low tension in the DME cells. As closure ensues, DME cells begin to accumulate F-actin and Myosin at the leading edge to form the supracellular contractile actomyosin cable (Young et al., 1993; Kiehart et al., 2000; Jacinto et al., 2002; Kaltschmidt et al., 2002), and the leading edge smoothens into a neatly organized row of DME cells. Co-incident with this transition, the DME cells elongate in the dorsoventral direction (Figure 2A) (Kiehart et al., 2000). We did not find any obvious difference in the shapes of DME or lateral epidermal cells between stage 13 control (*n =* 24) and SJ mutant animals (*n =* 15 for *Mcr* ^*1*^, *n =* 30 for *cora* ^*4*^, and *n =* 11 for *Nrx-IV* ^*4304*^) (Figures 2D,G,J). Beginning in stage 14, the lateral epidermal cells in *w* ^*1118*^ embryos elongate along their dorsoventral axis as the epidermal sheets spread to cover the dorsal surface, and the leading edge of the DME cells adopt a smooth and taught appearance (Figure 2B,C). In our analysis, only 12.5% of stage 14 *w* ^*1118*^ embryos (*n =* 24) showed mild irregularities at the leading edge and none had any visible defects in epidermal elongation. In contrast, stage 14 *Mcr* ^*1*^, *cora* ^*4*^, and *Nrx-IV* ^*4304*^ embryos had frequent mild irregularities in the appearance of the leading edge and substantial defects in dorsoventral elongation of the lateral epidermal cells (Figures 2E,H,K). Specifically, 72.7% of *Mcr* ^*1*^ (*n =* 11), 83.8% of *cora* ^*4*^ (*n* = 31), and 76.5% of *Nrx-IV* ^*4304*^ (*n =* 17) mutant embryos possessed distinct groups of lateral epidermal cells that failed to elongate in the dorsoventral axis and instead appeared wider along the anterior-posterior axis compared to surrounding cells. By late stage late 14/early stage 15, every SJ mutant embryo showed clear examples of defective cell elongation. In 92.3% of *Mcr* ^*1*^ (*n* = 13), 93.5% of *cora* ^*4*^ (*n =* 30), and 94.4% of *Nrx-IV* ^*4304*^ (*n* = 18) embryos, the leading edge appeared scalloped. In all these embryos, groups of cells at the leading edge and the more ventral row of cells failed to elongate as compared to *w* ^*1118*^ embryos (*n* = 33) (Figures 2C,F,I,L). Most of these cells appeared larger, rounder or had irregular cell boundaries. Some of the stage 14 and stage 14/15 SJ mutant embryos exhibited even more severe defects, including those that displayed a large number of epidermal cells failing to elongate, bunching of groups of cells at the leading edge, or even visible tearing along the leading edge (Supplementary Figure S3).

**FIGURE 3:**
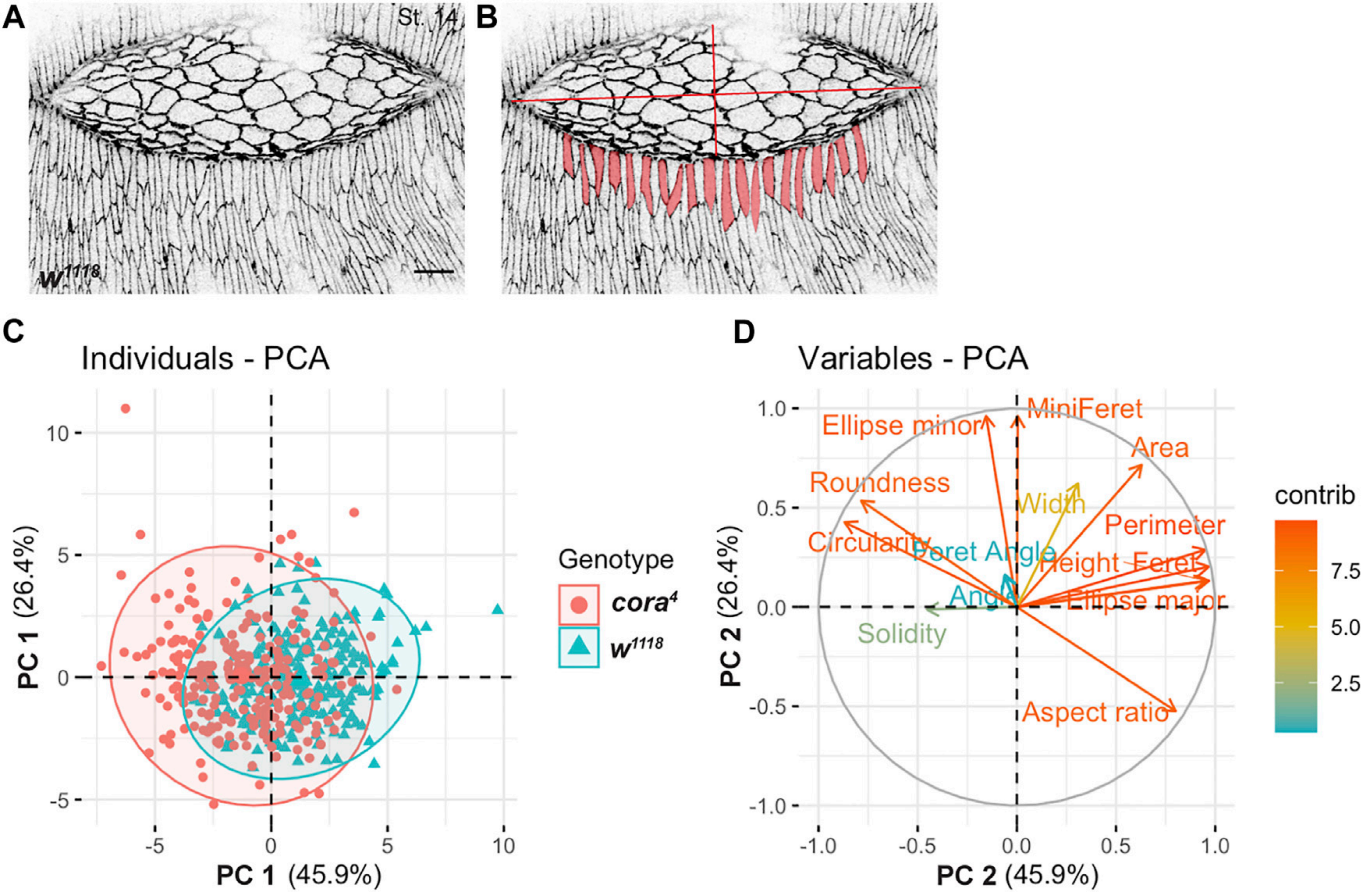
Principal Component Analysis (PCA) of cell shape. **(A)** Confocal optical section of a stage 14 wild type embryo stained with antibody against Ecad. **(B)** Segmented views of leading edge cells subjected to Fiji-based morphometric measurements. **(C)** Projection of individual cell shape measurements of lateral epidermal cells in stage 14 *w* ^*1118*^ (cyan) and *cora* ^*4*^ (red) across the two major principal components 1 (PC 1) and 2 (PC 2) revealed a large overlap with some regions of separation. **(D)** Contribution of 14 2D cell shape variables to the two principal components (on a scale of 0–12) showed a greater influence of height, ellipse major, perimeter, aspect ratio, roundness and circularity on PC 1 and ellipse minor and area on PC 2. Scale bar = 10 µm.

To quantify these cell shape defects, we performed a morphometric analysis of the lateral epidermal cells in stage 14 *w* ^*1118*^ and *cora* ^*4*^ embryos that had similar sized dorsal holes (*n =* 40 cells per embryo, *n* = 6 embryos per genotype). We used Fiji to segment and quantify 14 2D cell shape descriptors of leading edge and lateral epidermal cells, including height (length along DV axis), width (length along AP axis), aspect ratio, perimeter, and area (Figures 3A,B; Table 1). We performed a principal component analysis to determine the most important cell shape variables that contribute to maximum variance between *cora* ^*4*^ and control cells. The first two principal components (PC 1 and PC 2) generated accounted for 45.9% and 26.4% of the total variance, respectively (Figure 3C). A correlation plot of the contribution of cell shape descriptors to the variance showed that *cora* ^*4*^ cells differ from control cells due to a major positive influence of height, ellipse major (primary axis of the best fitted ellipse), aspect ratio (major to minor axis of best fitted ellipse), and perimeter on PC 1. Ellipse minor (secondary axis of the best fitted ellipse) and area had a greater positive influence in the direction of PC 2 (*p* < 0.05) (Figure 3D). Together, these descriptors indicate that DME and lateral epidermal cells in *cora* ^*4*^ mutant embryos are defective in elongating in the dorsoventral axis and likely fail to flatten in the apical-basal axis.

**TABLE 1:** Quantification of DC defects in cuticle preparations of *cora* ^*4*^ *and cora* ^*4*^ *, shg* ^*2*^ */cora* ^*4*^ *, +* embryos.

Genotype	% DC defects
*cora* ^*4*^	56.74%
*cora* ^*4*^ *, shg* ^*2*^ */cora* ^*4*^ *, +*	70.1%^****^

All values are absolute percentages of embryos with dorsal open phenotypes in cuticle preparations; *n =* 356 for *cora* ^*4*^ mutant embryos; *n =* 398 for *cora* ^*4*^ *, shg* ^*2*^ */cora* ^*4*^ *, +* mutant embryos; ^****^exact and asymptotic *p*-value = 0.0001 in a two-by-two analysis*.*

**FIGURE 4:**
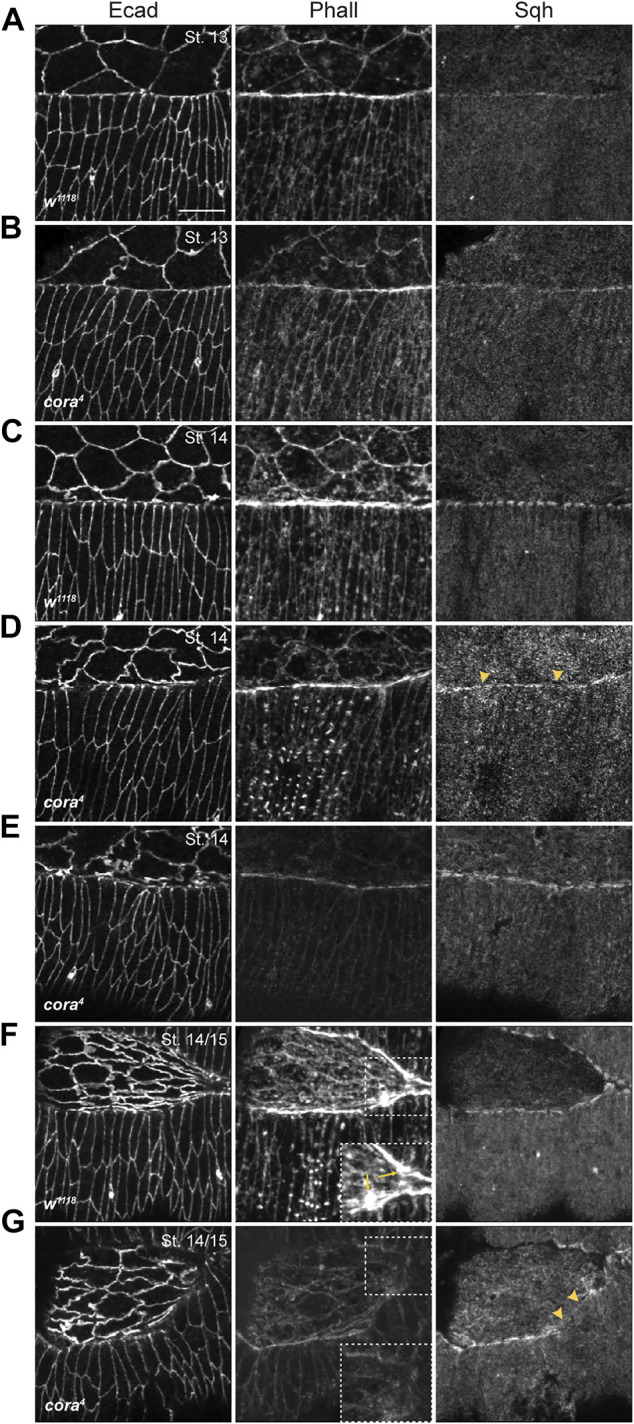
SJ mutant embryos have defects in actomyosin cable formation during late stages in DC. **(A–G)** Confocal optical sections of stage 13, stage 14, and stage14-15 wild type and *cora* ^*4*^ embryos stained with antibodies against Ecad, Sqh and Alexa Fluor 555 Phalloidin (Phall). **(A)** Stage 13 wild type embryos have continuous enrichment of F-actin and mild accumulation of Sqh at the leading edge. **(B)** *cora* ^*4*^ embryos exhibit a similar expression of Sqh and F-actin at the leading edge, with mild reduction in F-actin enrichment. **(C)** The leading edge of stage 14 wild type embryos have strong enrichment of F-actin, along with a “bars-on-string” distribution of Sqh. **(D)** Stage 14 *cora* ^*4*^ embryos have a reduction of F-actin at the leading edge. Sqh enrichment at the leading edge is discontinuous with regions of diffused expression (yellow arrowheads). **(E)** Some stage 14 *cora* ^*4*^ embryos exhibit dramatic reduction in F-actin accumulation at the leading-edge, epidermis, and amnioserosa. **(F)** Stage 14-15 wild type embryos continue to accumulate F-actin and Sqh at the leading edge, with stronger protrusive expression near to the canthi that are prominent in the zoomed in regions (yellow arrows in dashed box). **(G)** In contrast, the leading edge, lateral epidermis and amnioserosa of stage 14-15 *cora* ^*4*^ embryos have a severe reduction in F-actin expression, with no visible protrusive expression (dashed box showing zoomed in region) along with diffused and discontinuous Sqh distribution (yellow arrowheads). Scale bar = 10 µm.

Defects in the actomyosin cytoskeleton follow a similar pattern of progressive decay in SJ mutant embryos. To examine actomyosin cable formation and maintenance in the leading edge of DME cells we hand devitellinized *w* ^*1118*^, *cora* ^*4*^, *Mcr* ^*1*^, and *Nrx* ^*4304*^ mutant embryos and stained them with Alexa 555-labeled phalloidin and antibodies against Spaghetti squash (Sqh; Figure 4 and Supplementary Figure S4). Phalloidin staining in stage 13 *w* ^*1118*^ embryos (*n =* 8) revealed expected F-actin enrichment at the leading edge of DME cells, along with cortical F-actin localization in the lateral epidermis and amnioserosa (Figure 4A). Similarly, stage 13 *cora* ^*4*^ embryos (*n =* 9) accumulate F-actin at the leading edge of DME cells and in the cortices of amnioserosa and lateral epidermal cells (Figure 4B). In some *cora* ^*4*^ mutant embryos, however, the enrichment of F-actin at the leading edge appears less robust that in control embryos (Figure 4B). We found no differences in the expression of Sqh at the leading edge of DME cells or in the amnioserosa in stage 13 *cora* ^*4*^ animals compared to control embryos (Figures 4A,B). We also noted Sqh localization in the cytoplasm of amnioserosa cells in both *w* ^*1118*^ and *cora* ^*4*^ mutant embryos (Figures 4A,B). Taken together, these results again indicate that the early events of DC are relatively undisturbed in SJ mutant embryos.

Beginning in stage 14, however, SJ mutant embryos showed clear defects in the expression and localization of F-actin and Myosin. In stage 14 *w* ^*1118*^ embryos (*n =* 8), the leading edge continued to accumulate F-actin (Figure 4C) along with the amnioserosa and lateral epidermis. At this stage, Sqh distribution at the leading edge had a characteristic “bars-on-string” appearance. In contrast, *cora^4^* embryos (n = 15) exhibited a reduction in F-actin localization at the leading edge and the cortical distribution of F-actin in the lateral epidermis and amnioserosa appeared reduced compared to control embryos (Figure 4D). Similarly, Sqh distribution was diffuse and lacked a robust “bars-on-string” pattern in *cora^4^* mutant embryos, although its localization in the epidermis and amnioserosa appeared unaffected (Figure 4D). We observed that 33% of these *cora^4^* embryos had severe loss of F-actin from the leading edge, epidermis and amnioserosa (Figure 4E).

Towards the end of closure in stage 14/15 *w* ^*1118*^ embryos (*n* = 10), persistence of Sqh and F-actin enrichment at the leading edge was accompanied by appearance of F-actin-rich protrusions near the canthi (Figure 4F). In contrast, stage 14/15 *cora* ^*4*^ animals (*n* = 9) had a nearly complete loss of F-actin accumulation at the leading edge, lateral epidermis and amnioserosa (Figure 4G). We also failed to observe any visible F-actin-rich protrusions at the leading edge or the canthi in these embryos. Sqh distribution was found to be diffuse at the leading edge with small regions of complete loss, although Sqh localization in the epidermis and amnioserosa appeared to be unaffected (Figure 4G).

Stage 14 and stage 14/15 *Mcr* ^*1*^ and *Nrx-IV* ^*4304*^ animals exhibited defects in F-actin and Sqh localization at the leading edge, lateral epidermis and amnioserosa, similar to that observed in *cora* ^*4*^ embryos (Supplementary Figure S4). Together these results indicate a requirement of SJ proteins in maintaining actomyosin distribution in the dorsal epidermis and amnioserosa late in closure.

### The epidermis tears free from the amnioserosa during late DC in SJ mutant embryos

**FIGURE 5:**
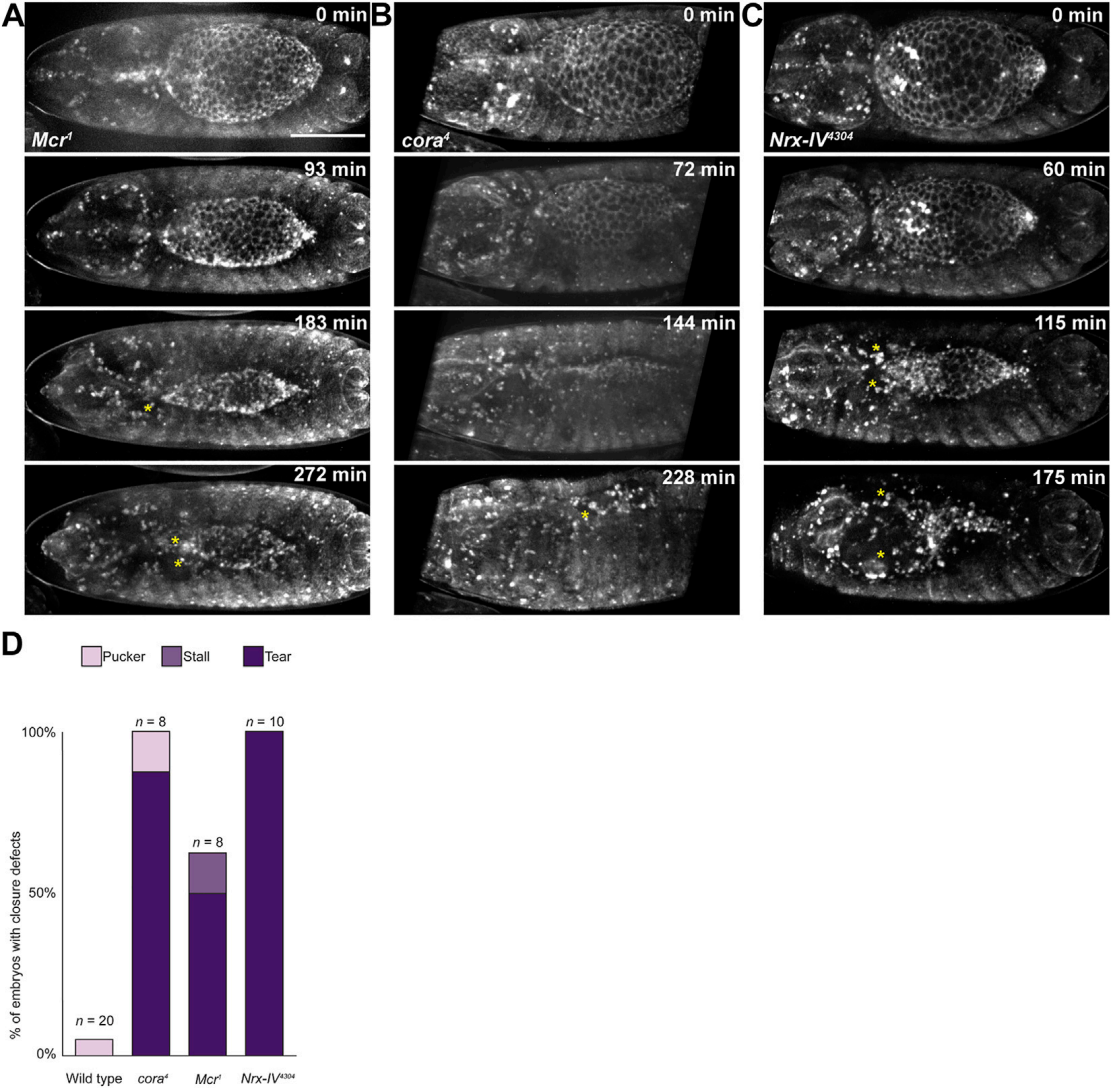
Late in DC, the epidermis tears away from the amnioserosa in SJ mutant embryos. **(A–C)** Still images from time lapse series of SJ mutant embryos during closure. Yellow stars indicate tears. **(D)** Observed frequency of closure defects in wild type and SJ mutant embryos. Scale bar = 100 µm.

We noted examples of SJ mutant embryos with clear separations between the DME cells and the amnioserosa from fixed tissue staining (Supplementary Figure S2). Live imaging analyses using the tdTomato-tagged *shg* knock-in allele confirmed that these tears occurred as part of the closure process and were not artifacts of fixation and staining (Figures 5A–C). Tearing was not observed in *w* ^*1118*^ embryos but occurred in all three SJ mutant lines at varying penetrance—87.5% (*n* = 8) among *cora* ^*4*^ embryos, 50.0% (*n =* 8) among *Mcr* ^*1*^ embryos, and 100.0% (*n* = 10) among *Nrx-IV* ^*4304*^ embryos (Figure 5D). Tearing tended to occur earlier in *Nrx-IV* ^*4304*^ embryos than in *cora* ^*4*^ or *Mcr* ^*1*^, typically shortly after the dorsal hole length dropped below 190 μm, leading to larger tears. In addition, tearing occurred more often on the anterior half of the dorsal hole. Since tearing happened late in DC when the rate of closure was slower in SJ mutants than in control embryos and cell shape changes are aberrant, we wanted to determine if the lateral epidermis is under increased tension in these SJ mutant embryos or if adhesive defects were responsible for this phenotype.

**FIGURE 6:**
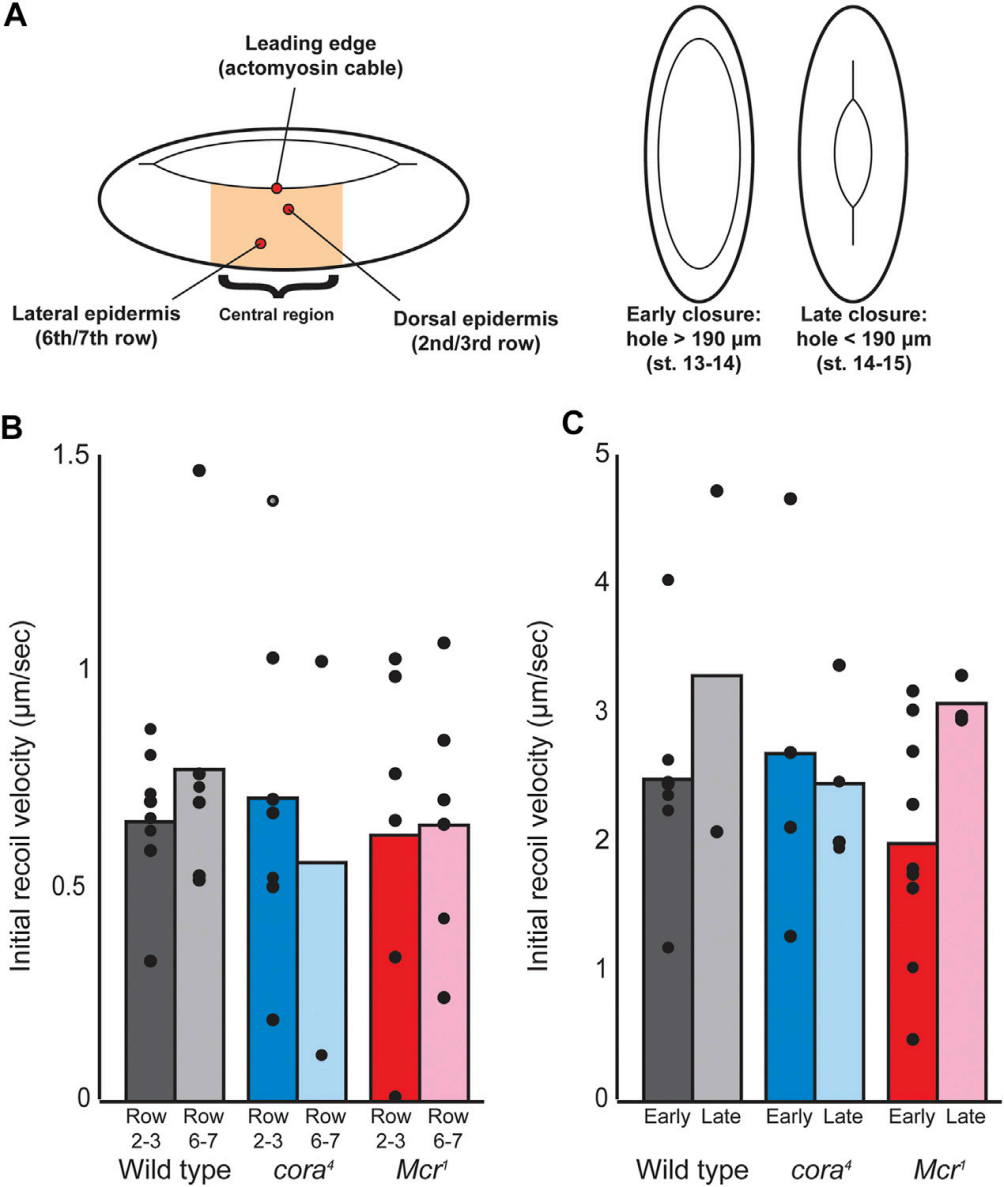
Laser ablation of cell junctions during dorsal closure. **(A)** Laser ablation scheme. Single cell junctions were ablated within the central region of the embryo during dorsal closure at three different locations, including the dorsal epidermis (second or third row of cells from the leading edge), lateral epidermis (sixth or seventh row of cells from the leading edge), and actomyosin cable (along the leading edge), and both early and late timepoints. Timepoints were determined based on the dorsal hole length (canthus to canthus), with dorsal holes > 190 μm considered “early” and dorsal holes < 190 μm considered late. **(B)** Initial recoil velocity of tricellular junctions neighboring the cut junction at both dorsal and lateral epidermal regions during late closure. No significant differences were observed either across genotypes or across locations. **(C)** Initial recoil velocity of tricellular junctions neighboring cell junctions cut along the leading edge at both early and late timepoints. Differences between genotypes and between timepoints were not significant.

The lateral epidermis of *Drosophila* embryos during dorsal closure is under tension that pulls the tissue away from the dorsal hole, resisting closure (Kiehart et al., 2000; Hutson et al., 2003). We reasoned that if SJ proteins are involved in transmitting forces across the tissue, this tension may be altered in SJ mutants, resulting in tearing. To test this, we used laser ablation to cut transverse cell junctions in the lateral epidermis in both dorsal regions of the epidermis (two to three rows from the leading edge) and more ventral regions (six to seven rows from the leading edge), calculating relative tension based on the initial rate of retraction (recoil velocity) of neighboring cell junctions as previously described (Zulueta-Coarasa and Fernandez-Gonzalez, 2015) (Figure 6A). We found no significant difference in tension between *w* ^*1118*^ and SJ mutant embryos in either location during early or late dorsal closure (Figure 6B). The actomyosin “cable” that runs along the leading edge in the DME cells is also under tension and plays a role in DC, either by acting as a “purse string” or maintaining a smooth and organized leading edge (Kiehart et al., 2000; Wood et al., 2002; Ducuing and Vincent, 2016). This cable is discontinuous (Ducuing and Vincent, 2016), and we reasoned that SJ proteins may be involved in transmitting tension along it, so we also used laser ablation to measure relative tension along the cable. We found no difference in tension between control and SJ mutant embryos during either early or late closure (Figure 6C). We also compared the time taken for the neighboring cell junctions to reach maximum displacement after junction ablation, which can be used to infer the viscosity-elasticity ratio of a tissue. Again, we found no differences between control and mutant embryos (data not shown). Therefore, we conclude that the tearing observed in SJ mutant embryos does not result from altered tension or tissue mechanics in the lateral epidermis of these embryos during closure.

**FIGURE 7:**
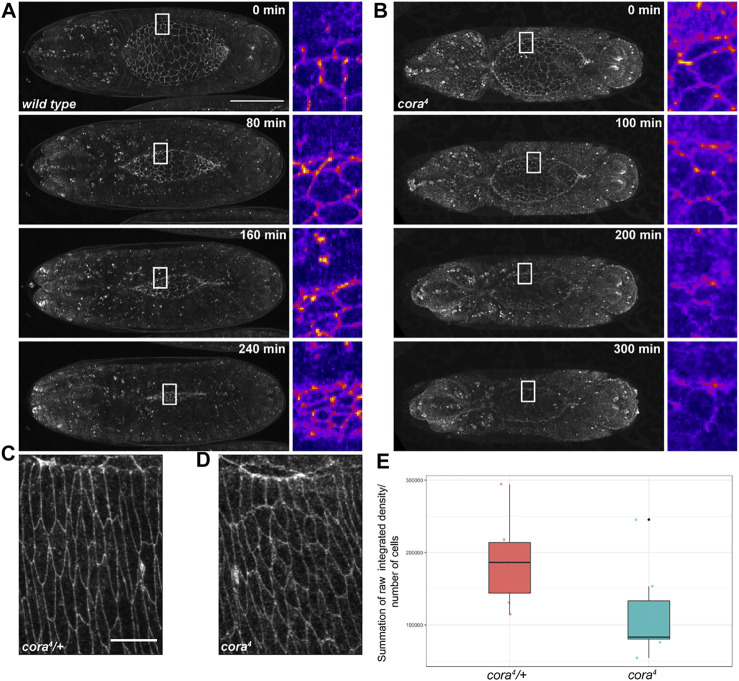
E-cadherin is depleted in SJ mutant embryos. **(A,B)** Still images from confocal time series during dorsal closure of wild type and *cora* ^*4*^ mutant embryos expressing tdTomato-tagged E-cadherin. E-cadherin fluorescence marking the edge of the dorsal hole and amnioserosa cell junctions is maintained and even brightened over the course of closure in wild type embryos but fades in the region around the dorsal hole in septate junction mutants. White rectangles indicate regions shown in zoomed out images, which are shown using Fiji’s “Fire” lookup table. **(C,D)** Z-projection of summed slices from zoomed in regions of z-series of stage 14 *cora* ^*4*^ */+* and *cora* ^*4*^ embryos stained with an antibody against E-cadherin. Note: the z-projection of *cora* ^*4*^ embryos has more cells than the control. **(E)** Boxplot showing the distribution of normalized total pixel intensity values for stage 14 *cora* ^*4*^ */+* (in red) and *cora* ^*4*^ embryos (in cyan). The mean normalized raw integrated density for *cora* ^*4*^ */+* was 186440.8 pixels/cell, whereas the mean value for *cora* ^*4*^ animals was113008.1 pixels/cell, which was statistically different from *cora* ^*4*^ */+* embryos (*p* < 0.05; bootstrap approach). All values are summation of raw integrated density/number of cells ± SD of selected slices from z-series of stage 14 embryos with similar length (<170 µm) of the dorsal hole; *n* = 8 per genotype. Scale bar in (A) = 100 μm; scale bar in (C) = 10 µm.

Since the tearing that occurs in SJ mutant embryos does not appear to be caused by altered tissue mechanics, we reasoned that it may result from decreased adhesion between the lateral epidermis and amnioserosa during closure. We noticed a clear difference in E-cadherin expression between SJ mutant and control embryos in our live imaging studies (Supplemental Movies S1-4). In all control and SJ mutant embryos, cellular junctions in the lateral epidermis, amnioserosa, and along the leading edge of DME cells clearly label with E-cadherin during early stages of closure. However, starting around the time that the shortening of the dorsal hole begins to accelerate, E-cadherin expression in *w* ^*1118*^ and SJ mutant embryos begins to diverge. In control embryos, the E-cadherin signal appears to become brighter as closure proceeds, ultimately forming a bright band of expression in recently zippered regions before fading away 30–60 min after closure (Figure 7A). In contrast, E-cadherin expression begins to fade away from the dorsal hole region in SJ mutant embryos at this stage of closure, including from the cellular junctions of the amnioserosa and leading edge, often to the point where it becomes difficult to determine the exact boundaries of the dorsal hole (Figure 7B). Examination of E-cadherin staining in fixed tissues samples supported this observation (Figure 7 and Supplementary Figure S5). Specifically, we stained embryos from an overnight collection of *cora* ^*4*^ */+* heterozygous adults with antibodies against E-cadherin and imaged stage 14 *cora* ^*4*^ mutant and *cora* *^4^*/+ heterozygous embryos using identical settings. We then totaled pixel intensities from all sections of z-series that encompassed the lateral epidermis and divided by the total number of cells, normalizing E-cadherin expression by cell. At stage 14, there is significantly less total E-cadherin per cell in *cora* ^*4*^ mutant embryos compared to *cora^4^*/+ heterozygous control embryos (Figure 7E). Interestingly, the E-cadherin that is present in *cora* ^*4*^ mutant cells also appears more diffuse and cytoplasmic than in heterozygous control cells (Figures 7C,D). We wondered whether the reduced expression and diffuse staining of E-cadherin in *cora* ^*4*^ mutant embryos was specific for E-cadherin or if reflected the adherens junction more generally. We therefore stained stage 13–15 *cora* ^*4*^ mutant and *cora* ^*4*^ */+* heterozygous control embryos with antibodies against E-cadherin and Armadillo (Supplementary Figure S6) or E-cadherin and α-catenin (not shown) and examined them by confocal microscopy. We observed similar results in both cases in which early mutant embryos (dorsal hole >190 μm) had robust expression of all adherens junctions proteins similar to that of control embryos (not shown), whereas late mutant embryos (dorsal hole <190 μm) showed generally lower expression of E-cadherin, Armadillo and α-catenin than control embryos with more cytoplasmic puncta (Supplementary Figure S6 and data not shown).

To address whether changes in adhesion may affect DC, we recombined an amorphic allele of E-cadherin (*shg* ^*2*^) onto the *cora* ^*4*^ chromosome and compared the penetrance of DC defects in *cora* ^*4*^ mutant embryos that were heterozygous for a mutation in E-cadherin with those without the E-cadherin mutation (Table 1). The *shg* ^*2*^ allele has been shown to have no effect on DC in heterozygous embryos and was used previously to address the effect of adhesion on other protein complexes during DC (Razzell et al., 2018). The experiment was done in triplicate and a total of 202 out of 356 *cora* ^*4*^ mutant embryos displayed a dorsal open phenotype. In contrast, 279 out of 398 *cora* ^*4*^ *, shg* ^*2*^ */cora* ^*4*^ *, +* mutant embryos were dorsal open. Cuticle phenotypes were treated as proportions and statistical significance was determined by comparing difference in the probability of the dorsal open phenotype in a population. A two-by-two analysis generated an exact and an asymptotic *p*-value of 0.0001, indicating a significant difference in the proportion of cuticle preparations with a dorsal open phenotype between *cora* ^*4*^ and *cora* ^*4*^ *, shg* ^*2*^ */cora* ^*4*^, + embryos. Together, these results indicate a role for cell adhesion in the DC defects associated with *cora* ^*4*^ mutant embryos.

## Discussion

In this study, we demonstrate a role for a subset of core SJ genes during dorsal closure in *Drosophila*. Through live and fixed tissue imaging, we reveal that *cora*, *Nrx-IV*, and *Mcr* are required late in DC to maintain robust actomyosin structures in the epidermis, for optimal cell shape changes necessary for the contralateral epidermal sheets to meet at the dorsal midline, and to maintain adhesion between the lateral epidermis and amnioserosa. The striking similarity in the cellular phenotypes associated with mutations in all three genes suggests that they are functioning together, much like they function together in providing an occluding junction, and raises the possibility that other SJ genes may also be required for these steps in DC. It should be emphasized that these events in DC are occurring before the SJ is structurally and physiologically intact, suggesting that the function of these proteins in morphogenesis is independent of their occluding function at the junction.

SJ genes do not appear to be required for the initiation or early events of DC. In SJ mutant embryos, JNK signaling is activated and the DME cells produce an actomyosin cable at the leading edge and elongate towards the dorsal midline (Figures 2, 4 and Supplementary Figure S2). The rate of closure during the first 60–90 min of DC in mutant embryos is similar to that observed in wild type embryos (Figure 1). Defects in SJ mutant embryos become apparent at stage 14 coincident with the time that wild type embryos enter the fast phase of DC. At this stage, SJ mutant embryos fail to enter the fast phase of closure and begin to display aberrant cell shapes (Figure 2). Cell shape defects become increasingly more prevalent and severe as closure continues. Actomyosin levels at the leading edge and in cell cortices are gradually reduced in mutant embryos, and discontinuities arise in the leading edge cable of some of these embryos (Figure 4). F-actin-based protrusive structures in DME cells are noticeably reduced in SJ mutant embryos, suggesting defective zippering in the mutants, although additional live imaging will be required to determine the magnitude and dynamics of these defects. Finally, the epidermis tears free from the amnioserosa in a substantial percentage of mutant embryos, producing a hole (Figure 5). Interestingly, the penetrance of this tearing phenotype appears to be higher than the penetrance of dorsal open cuticles suggesting that some of these embryos may repair using zippering from the canthi.

SJ protein function is required for cell shape changes late in DC. We observed normal dorsoventral elongation of DME cells during initiation of DC in SJ mutant embryos, followed by elongation of the lateral epithelial cells during stage 13 and 14 of embryogenesis (Figure 2). We noticed the appearance of misshapen cells that occurred singly or in small clusters in stage 14 mutant embryos. These defects became more apparent in later stage mutant embryos, but still showed examples of individually misshapen cells surrounded by more uniformly elongated cells, suggesting that these defects are mosaic and may arise cell autonomously. Morphometric analysis of DME and lateral epidermal cells from wild type and *cora* ^*4*^ mutant stage 14 embryos revealed significant differences in several cell shape descriptors (Figure 3). The descriptors we found most telling are height, which is the elongation along the dorsoventral axis, aspect ratio, and surface area (near the apical surface as we used E-cadherin to label the cells). It seems clear that some *cora* ^*4*^ mutant cells fail to maintain an elongated state. Although we found no evidence that there are tension or viscoelastic differences between wild type and *cora* ^*4*^ mutant cells (Figure 6), it is possible that there are local differences in tension that contributed to these defects. More interestingly, if the volume of wild type and *cora* ^*4*^ mutant cells are equivalent, *cora* ^*4*^ mutant cells are approximately 25% taller along the apical-basal axis than wild type cells at this stage of development. This observation raised an obvious, but underappreciated, aspect of DC; namely, that in order for a fixed number of lateral epidermal cells to cover the surface area of the amnioserosa (estimated to be approximately 40% of the surface area of the embryo at the start of DC), the cells must undergo a substantial contraction in the apical-basal axis. Our morphometric analysis investigated embryos in which the dorsal hole has a length of 65–150 μm and thus was well into the fast phase of DC in the wild type embryos. We wonder if cell flattening of lateral epidermal cells is developmentally-regulated during DC, and whether this process may contribute to the difference between the slow and fast phase of DC. Additional studies should be directed at answering this question.

SJ protein function is required late in DC to maintain adhesion between the AS and the epidermis. Given the high penetrance of tearing that we observed in *Nrx-IV* ^*4304*^, *cora* ^*4*^, and *Mcr* ^*1*^ mutant embryos (Figure 5), we wondered whether SJ mutant epidermal tissue was under higher tension, or if it had less adhesion to the amnioserosa. Laser cutting experiments did not reveal significant differences in tension or viscoelastic properties during early or late stages of closure in DME cells or in epidermal cells several rows ventral to the leading edge (Figure 6), arguing against a role for SJ proteins in regulating tension. If anything, SJ mutant may experience less tension late in DC as F-actin and Myosin expression and localization show a progressive decay towards the end of closure (Figure 4), and studies of DC and wound healing in embryonic epithelia have revealed a correlation between loss of tension and reduced myosin levels (Ducuing and Vincent 2016; Kobb et al., 2017). It is possible that the location and timing of our laser ablation experiments failed to capture these defects.

In contrast, SJ protein function appears necessary for proper adhesion between the epidermis and amnioserosa during the later stages of DC. Adhesion between the epidermis and the amnioserosa comprises two different adhesive complexes: E-cadherin-based adherens junctions, which localize to the apical lateral region of the interface between the DME and amnioserosa, and integrin-based focal adhesions, which localize to a more basal region of this interface (Narasimha and Brown, 2004; Gorfinkiel and Arias, 2007). The geometry of this interface is somewhat unique in that amnioserosa cells adopt a wedge shape over which the DME cells spread, resulting in a diagonal junction with an increased surface area than would be expected given the overall apical-basal height of these cells (Narasimha and Brown, 2004). We quantified the length of these interfaces and found no difference between *cora* ^*4*^ mutant embryos and wild type controls (Supplementary Figure S7). Similarly, we observed no difference in expression level or localization of the β-integrin Myospheroid in *cora* ^*4*^ mutant embryos versus controls (data not shown). In contrast, live imaging revealed a clear reduction in td-Tomato E-cadherin levels at the dorsal hole (comprising both DME cells and the remainder of the visible amnioserosa cells) in SJ mutant embryos compared to wild type controls (Figure 7). In fact, it was often difficult to follow the end of closure in SJ mutant embryos because the tdTomato E-cadherin signal was so dim. Immunostaining of fixed tissues supported this observation and suggested that E-cadherin-based adhesion may have been compromised late in closure (Figure 7 and Supplementary Figure S5). We reasoned that if SJ protein-dependent reduction of E-cadherin is a major contributor to the tearing phenotype, then further reducing endogenous E-cadherin should exacerbate the phenotype and increase the penetrance of DC defects. This hypothesis was borne out by a significant increase in cuticles with DC defects in *cora* ^*4*^ *shg* ^*2*^ */cora* ^*4*^ *+* versus *cora* ^*4*^ homozygous embryos (Table 1). A similar approach was used by Razzell and colleagues to demonstrate *ajuba*’s role in adhesion during DC (Razzell et al., 2018).

A potential complementary mechanism may involve a direct adhesive role for SJ proteins at the interface between the amnioserosa and epidermis. Many SJ proteins contain adhesive extracellular domains including Neuroglian, Gliotactin, Contactin, and Lachesin (Genova and Fehon, 2003; Faivre-Sarraih et al., 2004; Llimargas et al., 2004), and all of these proteins localize along the length of the lateral membrane during the time of DC (Hall and Ward, 2016). However, since SJ proteins are not expressed (or at least not at high levels) in the amnioserosa (Fehon et al., 1994; Moyer and Jacobs, 2008), it would seem likely that SJ proteins in the epidermis would have to form heterophilic interactions with as yet undetermined partners in the amnioserosa.

Embryonic wound healing has become an exceptional complementary model system to study biophysical and cellular responses during tissue morphogenesis. Our observations regarding the role of SJ proteins in DC are largely consistent with cellular responses to wound healing observed in SJ mutant embryos (Carvalho et al., 2018). In the study by Carvalho, the authors found that a significant percentage of embryos with mutations in eleven core SJ genes or genes required for assembly or localization of SJs failed to complete wound healing. They performed detailed cellular and biophysical analyses on late stage *kune-kune* (*kune*) mutant embryos. During wound healing in late-stage wild type embryos, cells at the edge of the wound assemble an actomyosin cable and close in approximately 1 h. In *kune* mutant embryos (categorized as strong mutants), the actin cable initially forms, but gradually reduces in intensity. Myosin levels show similar reductions in *kune* mutant embryos. Similar to our observations of DC in SJ mutant embryos, the rate of closure during wound healing is significantly slower in kune mutant embryos. During wound healing in in wild type embryos, E-cadherin levels at the wound edge are reduced, but this fold change in E-cadherin is significantly greater kune mutant embryos. Finally, Carvalho and colleagues showed that uninjured kune mutant embryos exhibited a range of cell shape difference in the smooth cells and denticle expressing cells of the ventral epidermis compared to wild type embryos. One important distinction between our studies and those in wound healing, however, is that during late stage wound healing the authors noted differences in viscoelastic properties between wild type and SJ mutant tissues. One possible explanation for this discrepancy is that at the time when wounds were induced, wild type tissues have a bona fide SJ that is lacking in the SJ mutants. It would not be surprising that a mature SJ may alter viscoelastic properties of the epidermis, and would differ from the tissue prior to its establishment or in tissue with mutations that prevent the formation of the junction. The control tissues in our study are wild type tissues prior to the formation of the junction and thus would not have experienced any changes associated with the formation of a mature SJ.

Taken together, these experiments reveal that SJ proteins are required late in DC to elicit and maintain proper cell shape, to maintain the expression levels and localizations of E-cadherin, F-actin and Myosin, and to maintain the adhesion between the epidermis and amnioserosa. One potential underlying mechanism that may unite these functions is a role for SJ proteins in maintaining polarity. A number of junctional and cytoskeletal proteins are planar polarized in DME cells during DC, and disruption of planar polarity leads to slower and disorganized closure (Kaltschmidt et al., 2002). Several SJ genes, including *cora*, have been implicated in planar polarity in pupal wings (Venema et al., 2004). Proper apical-basal polarity is also critical for efficient DC as disruption of the apical determinant Crumbs leads to defective DC characterized by aberrant cell shapes, loss of adhesion and enhanced actomyosin dynamics in the amnioserosa (Flores-Benitez and Knust 2015). Several SJ genes (including *cora*) have been implicated in a redundant pathway with *yurt* to maintain apical-basal polarity during these same stages of embryonic development (Laprise et al., 2010), and *yurt* is also required for DC (Hoover and Bryant, 2002). Perhaps SJ proteins serve a minor but underappreciated role in polarity on their own that is sufficient to lead to defect in DC when perturbed. Consistent with this notion, mutations in many SJ genes lead to long and convoluted trachea (e.g. Wu et al., 2007; Nelson et al., 2010; Batz et al., 2014), and this morphogenesis defect in *cora* mutant embryos can be rescued by reducing the level of Crb (Laprise et al., 2010). We expect that future studies will shed light on the role of SJ proteins in cell polarity and how this function contributes to efficient morphogenesis.

## Materials and methods

### *Drosophila* stocks

All fly stocks were maintained on media prepared with corn meal, sugar, yeast, and agar. Genetic experiments were performed in incubators maintained at 25°C. The SJ mutants used in these experiments were *cora* ^*4*^, *Mcr* ^*1*^, *Tsf* ^*KG01571*^, *Cont* ^*ex956*^, and *Nrx-IV* ^*4304*^ (Baumgartner et al., 1996; Ward et al., 1998; Faivre-Sarrailh et al., 2004; Tiklová et al., 2010; Hall et al., 2014). *cora* ^*4*^ and *Mcr* ^*1*^ mutants were balanced over *CyO, P{ActGFP.w* ^*-*^ *}CC2* or *CyO, P{w* ^*+*^ *, Gal4-twi.G}2.2, P{w* ^*+*^ *, UAS-2xEGFP}AH2.2*, while *Tsf* ^*KG01571*^, *Cont* ^*ex956*^, and *Nrx-IV* ^*4304*^ mutants were balanced over *TM6B, P{w* ^*+*^ *, Dfd-EYFP} or TM3, P{w* ^*+*^ *, Gal4-twi.G}2.3, P{UAS-2xEGFP}AH2.3, Sb* ^*1*^ *, Ser* ^*1*^ to allow identification of homozygous mutant embryos. *w* ^*1118*^, *cora* ^*4*^, *Nrx-IV* ^*4304*^, *Tsf* ^*KG01571*^, *shg^2^*, *puc* ^*E69*^ and balancer lines were obtained from the Bloomington *Drosophila* Stock Center (BDSC, Bloomington, IN). The tdTomato-tagged E-cadherin line (*shg*-tdTomato) was obtained from Rodrigo Fernandez-Gonzalez (University of Toronto).

### Immunostaining and imaging

Embryos were collected for 2–4 h or overnight and aged to the appropriate developmental stage at 25°C. Embryos were fixed and processed for antibody staining as described in Fehon et al. (1991). Detailed protocols and antibody dilutions are available at https://www.protocols.io/view/Immunohistochemistry-Drosophila-Embryo-cutwwm. For phalloidin staining, embryos were hand devitellinized using a tungsten needle followed by standard staining procedures. Antibodies against E-cadherin, Armadillo, alpha catenin, Myospheroid, Coracle, and β-galactosidase (LacZ) were obtained from the Developmental Studies Hybridoma Bank (University of Iowa, Iowa City, IA, United States), whereas antibody against Sqh was previously generated in our lab (Wang and Ward, 2010). FITC-labelled phosphotyrosine was obtained from Sigma. Alexa Fluor 555 Phalloidin was obtained from Cell Signaling Technology and used at 1:100. Secondary antibodies (Jackson ImmunoResearch Laboratories, West Grove, PA, United States) were used at 1:800. Confocal images were acquired on an Olympus FV1000 confocal microscope equipped with UPLSAPO 20X (0.85 NA) oil immersion lens, a 3i Olympus spinning disc confocal microscope using a LUCPlanFLN 40X (1.3 NA) oil immersion lens or a Leica Stellaris 5 confocal microscope with a 10x (0.40 NA) dry and a 63x (1.4 NA) oil immersion lens. Confocal z-series of the dorsal epidermis of stage 14-15 embryos were acquired when orientation of the embryo and curvature of the epidermis interfered with visibility of the leading edge in a single focal plane. Selected individual images from the stacks were then used to generate a maximum intensity projection in Fiji. Raw photomicrographs were cropped, rotated, and adjusted for brightness and contrast in Fiji (Schindelin et al., 2012). Figures were compiled in Adobe Illustrator 2020 (version 25.2.3).

### Live imaging and analysis

Cell outlines were visualized using tdTomato-tagged E-cadherin. Embryos were collected on apple juice agar plates for 2–4 h and aged an additional 8 h, then dechorionated in 6% sodium hypochlorite. Embryos at late germ band retraction or early dorsal closure stage were then mounted dorsal surface down on an open-face coverslip coated in heptane glue and attached to a Petri dish lid holder with a hole cut out, then immersed in halocarbon oil 700. Mutant embryos were identified by absence of GFP in the case of *cora* and *Mcr* or YFP in the case of *Nrx-IV.* Because *Dfd-EYFP* expression is not always visible at the onset of dorsal closure, absence of YFP expression was reconfirmed at the end of imaging. Images shown are projections of 8–20 planes and time points ranged from 10 to 12 min apart, acquired on an Olympus FV1000 confocal microscope equipped with a UPLSAPO 20X Oil (0.85 NA) and Fluoview software or a 3i Olympus spinning disc confocal microscope using a LUCPlanFLN 20X (0.45 NA). Embryos were imaged until closure or until they appeared to cease closing or tore. Dorsal hole length measurements were made by measuring from canthus to canthus. Traces in Figure 1B only include embryos for which imaging began when dorsal hole length > 220 μm. Early rate estimates were made starting from the last timepoint in which the dorsal hole length > 220 μm and ending at the first timepoint in which the dorsal hole length < 190 μm. Late rate estimates were made starting from the first timepoint in which the dorsal hole length < 190 μm and ending at the last timepoint before closure to ensure that the measurement did not include any time at which the hole was already closed. In all three mutant genotypes, none of the homozygous mutant embryos hatched into larvae. Images in Figures 1, 5 were adjusted for brightness and contrast to show morphology, but images in Figure 7 showing differences in brightness were not adjusted. Zoomed in regions are shown using the “Fire” lookup table in Fiji.

### Laser ablation and tension measurement

We conducted laser ablation using a pulsed Micropoint N2 laser tuned to 365 nm and images were captured on a Revolution XD spinning-disk confocal microscope (Andor) using a 60x (NA 1.35) oil immersion lens (Olympus) and an iXon Ultra 897 camera (Andor). Stacks were acquired immediately before and after ablation and every 3 s thereafter for 60 s. Images in which only a single cell junction were cut were analyzed using SIESTA v 4.0 (Fernandez-Gonzalez and Zallen, 2011). We measured recoil velocity (indicative of relative tensile forces) based on the displacement of vertices at the ends of severed junctions in the first frame captured after cutting. Viscosity-elasticity ratios were estimated using a Kelvin-Voigt model to represent junctions (Fernandez-Gonzalez et al., 2009). According to this model, the viscosity-to-elasticity ratio is given by the relaxation time for the vertex displacements after ablation. The relaxation time (τ) was calculated by fitting junction retraction to equation *L(t)* = *D*(1 – *e* ^*t/τ*^), where *L(t)* is the distance between vertices at time *t* after ablation, and *D* is the asymptotic distance retracted, proportional to the stress-to-elasticity ratio.

### Morphometrics and Quantification of cell shape

Cell outlines from fixed tissues were visualized with an antibody against E-cadherin. Confocal images of the lateral epidermis of stage 14 embryos were acquired with the Olympus FV1000 confocal microscope (Olympus America, Inc., Center Valley, PA United States) equipped with a 40X oil (1.3 NA) immersion lens and Fluoview software or the Leica Stellaris 5 confocal microscope (Leica Microsystems Inc., Buffalo Groove, IL), equipped with LAS X software and a 63x (1.4 NA) oil immersion lens. Images were cropped and rotated in Fiji. Length and width of the dorsal hole were used to select stage 14 wild type and *cora* ^*4*^ embryos that were at similar phase of closure. To generate cell outlines, apical surfaces of 20 cells from the leading edge and the adjacent row ventral to the leading edge were manually segmented using the “Freehand selection” tool and “Segmentation editor” plugin in Fiji. After adjusting threshold, binary masks were generated from the segmented images. Measurements for 14 cell shape descriptors (height, width, area, aspect ratio, circularity, roundness, ellipse major, ellipse minor, perimeter, angle, Feret, MiniFeret, Feret’s angle, and solidity) were extracted with the “Analyze Particles” function in Fiji. Statistical significance for each of the cell shape parameters was determined using a student’s *t*-test on R, with *p* < 0.05 (R Core Team, 2020). To reduce the dimensionality of the extracted measurements, a principal component analysis (PCA) was performed in R with the “factoextra” package (Kassambara and Mundt., 2020). A PCA score plot with the distribution of cell shape measurements across the first two principal components (PC 1 and PC 2) was generated using R. To display the contribution (on a scale of 0–12) of each cell shape descriptor to the first two principal components, a correlation circle plot of the variables was produced in R. All figures were produced using the “ggplot2” package in R (Wickham, 2016). Figures were compiled in Adobe Illustrator 2020 (version 25.2.3).

### Cuticle preparations

Embryos were collected on apple juice agar plates for 3–4 h at 25°C and aged overnight. Mutant embryos were then selected based upon the absence of balancer produced GFP or YFP. Nonhatched embryos 48 h after egg laying were dechorionated in 6% sodium hypochlorite, mounted in Hoyer’s medium on microscope slides, and cleared overnight at 50°C. For the experiments presented in Figure 1, all genotypes were masked by one author (OD) and blindly scored by another author (RW). The cuticles were imaged using bright field illumination on a Leica DM2500 LED optical upright microscope.

### Fluorescence intensity quantification

To quantify fluorescence intensity for E-cadherin expression, we acquired z-series of the dorsal epidermis of stages 13–15 fixed embryos (*n* = 8) stained with antibody against E-cadherin, using a Leica Stellaris five laser scanning confocal microscope and a 63x oil objective (NA 1.4) with a step size of 0.3 µm with the same laser intensity and gain settings for all embryos. We selected confocal z-series of embryos with a dorsal hole length in the range of 170 to 70 µm. From each of the z-series, nine slices were selected and cropped to cover regions that included the leading edge and three to four rows of epidermal cells with approximately 10–15 cells in each row. The areas of the cropped regions were same for all selected z-series. For each of the slices in the selected z-series, the values for raw integrated densities (total pixel values in the region of interest) were extracted by using the “Measure” tool in Fiji. Summation of the raw integrated density values were normalized to the number of cells covered in the selected regions for each embryo. A Bootstrap approach was used to calculate statistical significance in R Studio for *p* < 0.05.

## Data Availability

The original contributions presented in the study are included in the article/Supplementary Material, further inquiries can be directed to the corresponding author.

## Supplementary material

Supplementary Movie 1
**Live imaging of DC in wild type embryo.** Dorsal view of a *w^1118^* embryo expressing TdTomato E-cadherin. Images are projections of z-stacks (8-20 planes, 2.4 μm apart) taken with a time interval of 10 minutes on an Olympus spinning disc confocal microscope with a LUCPlanFLN 20X objective (0.45 NA).

Supplementary Movie 2
**Live imaging of DC in *Mcr^1^* embryo.** Dorsal view of an *Mcr^1^* mutant embryo expressing TdTomato E-cadherin. Images are projections of z-stacks (8-20 planes, 2.4 μm apart) taken with a time interval of 12 minutes on an Olympus spinning disc confocal microscope with a LUCPlanFLN 20X objective (0.45 NA).

Supplementary Movie 3
**Live imaging of DC in *cora^4^* embryo.** Dorsal view of a *cora^4^* embryo expressing TdTomato E-cadherin. Images are projections of z-stacks (8-20 planes, 2.4 μm apart) taken with a time interval of 10 minutes on an Olympus spinning disc confocal microscope with a LUCPlanFLN 20X objective (0.45 NA).

Supplementary Movie 4
**Live imaging of DC in *Nrx-IV^4304^* embryo.** Dorsal view of a *Nrx-IV^4304^* embryo expressing TdTomato E-cadherin. Images are projections of z-stacks (8-20 planes, 2.4 μm apart) taken with a time interval of 10 minutes on an Olympus spinning disc confocal microscope with a LUCPlanFLN 20X objective (0.45 NA).

## References

[B1] Batz T. Forster D. Luschnig S. (2014). The transmembrane protein macroglobulin complement-related is essential for septate junction formation and epithelial barrier function in Drosophila. Development 141, 899–908. 10.1242/dev.102160 24496626

[B2] Baumgartner S. Littleton J. T. Broadie K. Bhat M. A. Harbecke R. Lengyel J. A. (1996). A *Drosophila* Neurexin is required for septate junction and blood-nerve barrier formation and function. Cell 87, 1059–1068. 10.1016/S0092-8674(00)81800-0 8978610

[B3] Behr M. Riedel D. Schuh R. (2003). The claudin-like Megatrachea is essential in septate junctions for the epithelial barrier function in Drosophila. Dev. Cell 5, 611–620. 10.1016/S1534-5807(03)00275-2 14536062

[B4] Carvalho L. Patricio P. Ponte S. Heisenberg C. P. Almeida L. Nunes A. S. (2018). Occluding junctions as novel regulators of tissue mechanics during wound repair. J. Cell Biol. 217, 4267–4283. 10.1083/jcb.201804048 30228162 PMC6279375

[B5] Ducuing A. Vincent S. (2016). The actin cable is dispensable in directing dorsal closure dynamics but neutralizes mechanical stress to prevent scarring in the *Drosophila* embryo. Nat. Cell Biol. 18, 1149–1160. 10.1038/ncb3421 27749820

[B6] Faivre-Sarrailh C. Banerjee S. Li J. Hortsch M. Laval M. Bhat M. A. (2004). *Drosophila* contactin, a homolog of vertebrate contactin, is required for septate junction organization and paracellular barrier function. Development 131, 4931–4942. 10.1242/dev.01372 15459097

[B7] Fehon R. G. Dawson I. A. Artavanis-Tsakonas S. (1994). A *Drosophila* homologue of membrane-skeleton protein 4.1 is associated with septate junctions and is encoded by the coracle gene. Development 120, 545–557. 10.1242/dev.120.3.545 8162854

[B8] Fernandez-Gonzalez R. Simoes S. M. Röper J. C. Eaton S. Zallen J. A. (2009). Myosin II dynamics are regulated by tension in intercalating cells. Dev. Cell 17, 736–743. 10.1016/j.devcel.2009.09.003 19879198 PMC2854079

[B9] Fernandez-Gonzalez R. Zallen J. A. (2011). Oscillatory behaviors and hierarchical assembly of contractile structures in intercalating cells. Phys. Biol. 8, 045005. 10.1088/1478-3975/8/4/045005 21750365 PMC4782797

[B10] Flores-Benitez D. Knust E. (2015). Crumbs is an essential regulator of cytoskeletal dynamics and cell-cell adhesion during dorsal closure in Drosophila. Elife 4, e07398. 10.7554/eLife.07398 26544546 PMC4718732

[B11] Fogerson S. M. Mortensen R. D. Moore R. P. Chiou H. Y. Prabhu N. K. Wei A. H. (2020). Identifying key genetic regions for cell sheet morphogenesis on chromosome 2L using a Drosophila deficiency screen in dorsal closure. G3 GenesGenomesGenetics 10, 4249–4269. 10.1534/g3.120.401386 PMC7642946 32978263

[B12] Genova J. L. Fehon R. G. (2003). Neuroglian, Gliotactin, and the Na+/K+ ATPase are essential for septate junction function in Drosophila. J. Cell Biol. 161, 979–989. 10.1083/jcb.200212054 12782686 PMC2172966

[B13] Gorfinkiel N. Arias A. M. (2007). Requirements for adherens junction components in the interaction between epithelial tissues during dorsal closure in *Drosophila* . J. Cell Sci. 120, 3289–3298. 10.1242/jcs.010850 17878238

[B14] Hall S. Bone C. Oshima K. Zhang L. McGraw M. Lucas B. (2014). Macroglobulin complement-related encodes a protein required for septate junction organization and paracellular barrier function in *Drosophila* . Development 141, 889–898. 10.1242/dev.102152 24496625 PMC3912832

[B15] Hall S. Ward R. E. (2016). Septate junction proteins play essential roles in morphogenesis throughout embryonic development in *Drosophila* . G3 GenesGenomesGenetics 6, 2375–2384. 10.1534/g3.116.031427 PMC4978892 27261004

[B16] Hijazi A. Masson W. Augé B. Waltzer L. Haenlin M. Roch F. (2009). Boudin is required for septate junction organisation in Drosophila and codes for a diffusible protein of the Ly6 superfamily. Development 136, 2199–2209. 10.1242/dev.033845 19502482

[B17] Hoover K. B. Bryant P. J. (2002). Drosophila Yurt is a new protein-4.1-like protein required for epithelial morphogenesis. Dev. Genes, Evol. 212, 230–238. 10.1007/s00427-002-0231-6 12070613

[B18] Hou X. S. Goldstein E. S. Perrimon N. (1997). *Drosophila* Jun relays the Jun amino-terminal kinase signal transduction pathway to the Decapentaplegic signal transduction pathway in regulating epithelial cell sheet movement. Genes Dev. 11, 1728–1737. 10.1101/gad.11.13.1728 9224721

[B19] Huang J. Zhou W. Dong W. Watson A. Hong Y. (2009). Directed, efficient, and versatile modifications of the Drosophila genome by genomic editing. Proc. Natl. Acad. Sci. U. S. A. 106, 8284–8289. 10.1073/pnas.0900641106 19429710 PMC2688891

[B20] Hutson M. S. Tokutake Y. Chang M.-S. Bloor J. W. Venakides S. Kiehart D. P. (2003). Forces for morphogenesis investigated with laser microsurgery and quantitative modeling. Science 300, 145–149. 10.1126/science.1079552 12574496

[B21] Jacinto A. Wood W. Balayo T. Turmaine M. Martinez-Arias A. Martin P. (2000). Dynamic actin-based epithelial adhesion and cell matching during *Drosophila* dorsal closure. Curr. Biol. 10, 1420–1426. 10.1016/S0960-9822(00)00796-X 11102803

[B22] Jacinto A. Wood W. Woolner S. Hiley C. Turner L. Wilson C. (2002). Dynamic analysis of actin cable function during *Drosophila* dorsal closure. Curr. Biol. 12, 1245–1250. 10.1016/S0960-9822(02)00955-7 12176336

[B23] Kaltschmidt J. A. Lawrence N. Morel V. Balayo T. Fernández B. G. Pelissier A. (2002). Planar polarity and actin dynamics in the epidermis of *Drosophila* . Nat. Cell Biol. 4, 937–944. 10.1038/ncb882 12447392

[B24] Kassambara A. Mundt F. (2020). Extract and visualize the results of multivariate data analyses. R package version 1.0.7. Available at: https://CRAN.R-project.org/package=factoextra .

[B25] Kiehart D. P. Galbraith C. G. Edwards K. A. Rickoll W. L. Montague R. A. (2000). Multiple forces contribute to cell sheet morphogenesis for dorsal closure in *Drosophila* . J. Cell Biol. 149, 471–490. 10.1083/jcb.149.2.471 10769037 PMC2175161

[B26] Kobb A. B. Zulueta-Coarasa T. Fernandez-Gonzalez R. (2017). Tension regulates myosin dynamics during Drosophila embryonic wound repair. J. Cell Sci. 130, 689–696. 10.1242/jcs.196139 28202603

[B27] Laprise P. Paul S. M. Boulanger J. Robbins R. M. Beitel G. J. Tepass U. (2010). Epithelial polarity proteins regulate Drosophila tracheal tube size in parallel to the luminal matrix pathway. Curr. Biol. 20, 55–61. 10.1016/j.cub.2009.11.017 20022244 PMC2821987

[B28] Llimargas M. Strigini M. Katidou M. Karagogeos D. Casanova J. (2004). Lachesin is a component of a septate junction-based mechanism that controls tube size and epithelial integrity in the Drosophila tracheal system. Development 131, 181–190. 10.1242/dev.00917 14681183

[B29] Lord B. A. DiBona D. R. (1976). Role of the septate junction in the regulation of paracellular transepithelial flow. J. Cell Biol. 71, 967–972. 10.1083/jcb.71.3.967 993276 PMC2109773

[B30] Manning L. A. Perez-Vale K. Z. Schaefer K. N. Sewell M. T. Peifer M. (2019). The Drosophila Afadin and ZO-1 homologues Canoe and Polychaetoid act in parallel to maintain epithelial integrity when challenged by adherens junction remodeling. Mol. Biol. Cell 30, 1938–1960. 10.1091/mbc.E19-04-0209 31188739 PMC6727765

[B31] Millard T. H. Martin P. (2008). Dynamic analysis of filopodial interactions during the zippering phase of *Drosophila* dorsal closure. Development 135, 621–626. 10.1242/dev.014001 18184725 PMC2440488

[B32] Mortensen R. D. Moore R. P. Fogerson S. M. Chiou H. Y. Obinero C. V. Prabhu N. K. (2018). Identifying genetic players in cell sheet morphogenesis using a Drosophila deficiency screen for genes on chromosome 2R involved in dorsal closure. G3 GenesGenomesGenetics 8, 2361–2387. 10.1534/g3.118.200233 PMC6027880 29776969

[B33] Moyer K. E. Jacobs J. R. (2008). Varicose: A MAGUK required for the maturation and function of Drosophila septate junctions. BMC Dev. Biol. 8, 99. 10.1186/1471-213X-8-99 18847477 PMC2575209

[B34] Narasimha M. Brown N. H. (2004). Novel functions for integrins in epithelial morphogenesis. Curr. Biol. 14, 381–385. 10.1016/j.cub.2004.02.033 15028212

[B35] Nelson K. S. Furuse M. Beitel G. J. (2010). The Drosophila claudin kune-kune is required for septate junction organization and tracheal tube size control. Genetics 185, 831–839. 10.1534/genetics.110.114959 20407131 PMC2907205

[B36] Nilton A. Oshima K. Zare F. Byri S. Nannmark U. Nyberg K. G. (2010). Crooked, Coiled and Crimpled are three Ly6-like proteins required for proper localization of septate junction components. Development 137, 2427–2437. 10.1242/dev.05260510.1242/dev.052605 20570942 PMC2889608

[B37] Pasakarnis L. Frei E. Caussinus E. Affolter M. Brunner D. (2016). Amnioserosa cell constriction but not epidermal actin cable tension autonomously drives dorsal closure. Nat. Cell Biol. 18, 1161–1172. 10.1038/ncb3420 27749821

[B38] Paul S. M. Ternet M. Salvaterra P. M. Beitel G. J. (2003). The Na+/K+ ATPase is required for septate junction function and epithelial tube-size control in the Drosophila tracheal system. Development 130, 4963–4974. 10.1242/dev.00691 12930776

[B39] R Core Team R. (2020). R core Team. R: A language and environment for statistical computing. Vienna, Austria: R Foundation for Statistical Computing. Available at: http://www.R-project.org/http://www.R-project.org/ .

[B40] Razzell W. Bustillo M. E. Zallen J. A. (2018). The force-sensitive protein Ajuba regulates cell adhesion during epithelial morphogenesis. J. Cell Biol. 217, 3715–3730. 10.1083/jcb.201801171 30006462 PMC6168262

[B41] Rice C. De O. Alhadyian H. Hall S. Ward R. E. (2021). Expanding the junction: New insights into non-occluding roles for septate junction proteins during development. J. Dev. Biol. 9, 11. 10.3390/jdb9010011 33801162 PMC8006247

[B42] Riesgo-Escovar J. R. Hafen E. (1997). Drosophila Jun kinase regulates expression of decapentaplegic via the ETS-domain protein Aop and the AP-1 transcription factor DJun during dorsal closure. Genes Dev. 11, 1717–1727. 10.1101/gad.11.13.1717 9224720

[B43] Schindelin J. Arganda-Carreras I. Frise E. Kaynig V. Longair M. Pietzsch T. (2012). Fiji: An open-source platform for biological-image analysis. Nat. Methods 9, 676–682. 10.1038/nmeth.2019 22743772 PMC3855844

[B44] Sokolow A. Toyama Y. Kiehart D. P. Edwards G. S. (2012). Cell ingression and apical shape oscillations during dorsal closure in Drosophila. Biophys. J. 102, 969–979. 10.1016/j.bpj.2012.01.027 22404919 PMC3296024

[B45] Solon J. Kaya-Çopur A. Colombelli J. Brunner D. (2009). Pulsed forces timed by a ratchet-like mechanism drive directed tissue movement during dorsal closure. Cell 137, 1331–1342. 10.1016/j.cell.2009.03.050 19563762

[B46] Tepass U. Hartenstein V. (1994). The development of cellular junctions in the Drosophila embryo. Dev. Biol. 161, 563–596. 10.1006/dbio.1994.1054 8314002

[B47] Tiklová K. Senti K.-A. Wang S. Gräslund A. Samakovlis C. (2010). Epithelial septate junction assembly relies on melanotransferrin iron binding and endocytosis in *Drosophila* . Nat. Cell Biol. 12, 1071–1077. 10.1038/ncb2111 20935638

[B48] Toyama Y. Peralta X. G. Wells A. R. Kiehart D. P. Edwards G. S. (2008). Apoptotic force and tissue dynamics during Drosophila embryogenesis. Science 321, 1683–1686. 10.1126/science.1157052 18802000 PMC2757114

[B49] Venema D. R. Zeev-Ben-Mordehai T. Auld V. J. (2004). Transient apical polarization of Gliotactin and Coracle is required for parallel alignment of wing hairs in Drosophila. Dev. Biol. 275, 301–314. 10.1016/j.ydbio.2004.07.04010.1016/j.ydbio.2004.07.040 15501220

[B50] Wang X. Ward R. E. (2010). Sec61α is required for dorsal closure during Drosophila embryogenesis through its regulation of Dpp signaling. Dev. Dyn. 239, 784–797. 10.1002/dvdy.22219 20112345 PMC2975395

[B51] Ward R. E. Evans J. Thummel C. S. (2003). Genetic modifier screens in Drosophila demonstrate a role for Rho1 signaling in ecdysone-triggered imaginal disc morphogenesis. Genetics 165, 1397–1415. 10.1093/genetics/165.3.1397 14668390 PMC1462826

[B52] Ward R. E. IVLamb R. S. Fehon R. G. (1998). A conserved functional domain of *Drosophila* coracle is required for localization at the septate junction and has membrane-organizing activity. J. Cell Biol. 140, 1463–1473. 10.1083/jcb.140.6.1463 9508778 PMC2132682

[B53] Wickham W. (2016). Wickham, H. ggplot2: Elegant graphics for data analysis. New York: Springer. Available at: https://ggplot2.tidyverse.org .

[B54] Wood W. Jacinto A. Grose R. Woolner S. Gale J. Wilson C. (2002). Wound healing recapitulates morphogenesis in *Drosophila* embryos. Nat. Cell Biol. 4, 907–912. 10.1038/ncb875 12402048

[B55] Wu V. M. Schulte J. Hirschi A. Tepass U. Beitel G. J. (2004). Sinuous is a Drosophila claudin required for septate junction organization and epithelial tube size control. J. Cell Biol. 164, 313–323. 10.1083/jcb.200309134 14734539 PMC2172325

[B56] Wu V. M. Yu M. H. Paik R. Banerjee S. Liang Z. Paul S. M. (2007). Drosophila Varicose, a member of a new subgroup of basolateral MAGUKs, is required for septate junctions and tracheal morphogenesis. Development 134, 999–1009. 10.1242/dev.02785 17267446 PMC1955473

[B57] Young P. E. Richman A. M. Ketchum A. S. Kiehart D. P. (1993). Morphogenesis in *Drosophila* requires nonmuscle myosin heavy chain function. Genes Dev. 7, 29–41. 10.1101/gad.7.1.29 8422986

[B58] Zahedi B. Shen W. Xu X. Chen X. Mahey M. Harden N. (2008). Leading edge-secreted Dpp cooperates with ACK-dependent signaling from the amnioserosa to regulate myosin levels during dorsal closure. Dev. Dyn. 237, 2936–2946. 10.1002/dvdy.21722 18816840

[B59] Zulueta-Coarasa T. Fernandez-Gonzalez R. (2015). “Laser ablation to investigate cell and tissue mechanics *in vivo* ,” in Integrative mechanobiology: Micro and nano techniques in cell mechanobiology. Editors Sun Y. Kim D. Simmons C. (Cambridge University Press). 10.1017/cbo9781139939751.009

