# Supplementary material for: Septate junction proteins are required for cell shape changes, actomyosin reorganization and cell adhesion during dorsal closure in Drosophila
Source: Front Cell Dev Biol. 2022 Sep 27;10:947444. doi: 10.3389/fcell.2022.947444 (PMC9553006; doi:10.3389/fcell.2022.947444)
Supplement: Supplementary file 4 [file DataSheet1.docx]

**Supplemental Information**

**Supplementary Movie 1. Live imaging of DC in wild type embryo.** Dorsal view of a *w^1118^* embryo expressing TdTomato E-cadherin. Images are projections of z-stacks (8-20 planes, 2.4 μm apart) taken with a time interval of 10 minutes on an Olympus spinning disc confocal microscope with a LUCPlanFLN 20X objective (0.45 NA).

**Supplementary Movie 2. Live imaging of DC in *Mcr^1^* embryo.** Dorsal view of an *Mcr^1^* mutant embryo expressing TdTomato E-cadherin. Images are projections of z-stacks (8-20 planes, 2.4 μm apart) taken with a time interval of 12 minutes on an Olympus spinning disc confocal microscope with a LUCPlanFLN 20X objective (0.45 NA).

**Supplementary Movie 3. Live imaging of DC in *cora^4^*embryo.** Dorsal view of a *cora^4^* embryo expressing TdTomato E-cadherin. Images are projections of z-stacks (8-20 planes, 2.4 μm apart) taken with a time interval of 10 minutes on an Olympus spinning disc confocal microscope with a LUCPlanFLN 20X objective (0.45 NA).

**Supplementary Movie 4. Live imaging of DC in *Nrx-IV^4304^* embryo.** Dorsal view of a *Nrx-IV^4304^* embryo expressing TdTomato E-cadherin. Images are projections of z-stacks (8-20 planes, 2.4 μm apart) taken with a time interval of 10 minutes on an Olympus spinning disc confocal microscope with a LUCPlanFLN 20X objective (0.45 NA).

| Cell shape descriptor | Mean value for apical surfaces of leading-edge cells in *w^1118^* | Mean value for apical surfaces of cells ventral to the leading-edge cells in *w^1118^* | Mean value for apical surfaces of leading-edge cells in *cora^4^* | Mean value for apical surfaces of cells ventral to the leading-edge cells in *cora^4^* |
| --- | --- | --- | --- | --- |
| Height | 12.47±2.44 μm | 12.78±2.69 μm | 9.84±2.72 μm *^****^* | 9.34±2.1 μm *^****^* |
| Width | 3.67±1.93 μm | 3.08±1.34 μm | 3.28±1.39 μm *^ns^* | 3.03±1.32 μm *^ns^* |
| Aspect ratio | 6.1±1.62 | 6.61±1.73 | 4.84±2.01 *^****^* | 4.87±1.63 *^****^* |
| Area | 22.66±6.47 μm^2^ | 20.1±6.09 μm^2^ | 18.32±7.59 μm^2^ *^****^* | 15.65±5.18 μm^2^ *^****^* |
| Perimeter | 28.93±5.44 μm | 28.48±5.68 μm | 23.34±5.75 μm *^****^* | 21.72±4.73 μm *^****^* |
| Circularity | 0.34±0.07 | 0.32±0.07 | 0.43±0.12 *^****^* | 0.43±0.1 *^****^* |
| Roundness | 0.17±0.05 | 0.16±0.04 | 0.25±0.12 *^****^* | 0.23±0.07 *^****^* |
| Solidity | 0.86±0.07 | 0.87±0.07 | 0.87±0.07 *^ns^* | 0.88±0.06 *^ns^* |
| Ellipse Major | 13.05±2.66 μm | 12.75±2.5 μm | 10.21±2.88 μm *^****^* | 9.58±2.2 μm *^****^* |
| Ellipse Minor | 2.2±0.39 μm | 1.99±0.42 μm | 2.29±0.66 μm *^ns^* | 2.08±0.51 μm *^ns^* |
| Angle | 87.67±13.71° | 95.49±10.44° | 92.17±14.81° *^*^* | 95.42±13.55° *^ns^* |
| Feret | 13.01±2.56 μm | 13.14±2.71 μm | 10.31±2.73 μm *^****^* | 9.81±2.24 μm *^****^* |
| MiniFeret | 2.49±0.5 μm | 2.27±0.54 μm | 2.58±0.76 μm *^ns^* | 2.31±0.59 μm *^ns^* |
| Feret Angle | 88.57±16.33° | 97.5±11.52° | 93.72±18.83° *^*^* | 96.36±16.96° *^ns^* |

**Table S1. Quantification of cell shape descriptors of apical surfaces of epidermal cells in *w^1118^* and *cora^4^* embryos.**

All measurements are mean value ± SD of 20 leading-edge cells or cells ventral to the leading-edge in each embryo; *n* = 6 embryos per genotype*; ^*^ p* < 0.05; *^****^ p* < 0.0001*; ^ns^ p* > 0.05 (unpaired t-test)


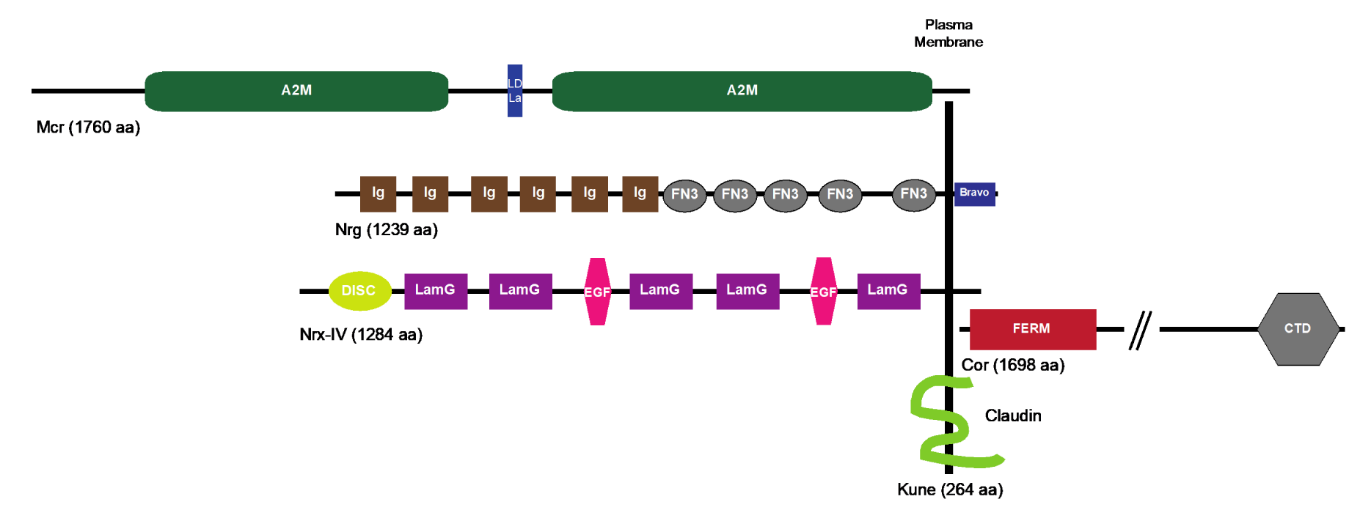


**Figure S1.** **Protein structures of representative core SJ proteins.** Diagrammatic representation of protein structures of core SJ proteins. Mcr, Nrg, Nrx-IV, and Kune are transmembrane proteins, whereas Cora is a cytoplasmic protein that binds to the intracellular domain of Nrx-IV. Nrg and Nrx-IV are adhesion molecules, whereas Kune is a claudin. A2M: Alpha-2-macroglubulin domain, LDLa: Low-density lipoprotein receptor class A repeat domain, Ig: Immunoglobulin domain, FN3: Fibronectin type 3 domain, Bravo: Bravo or NrCAM domain, DISC: Discoidin domain, LamG: Laminin G domain, EGF: Epidermal Growth Factor domain, FERM: Protein 4.1/Ezrin/Radixin/Moesin domain, CTD: Band 4.1 C-terminal domain.

**
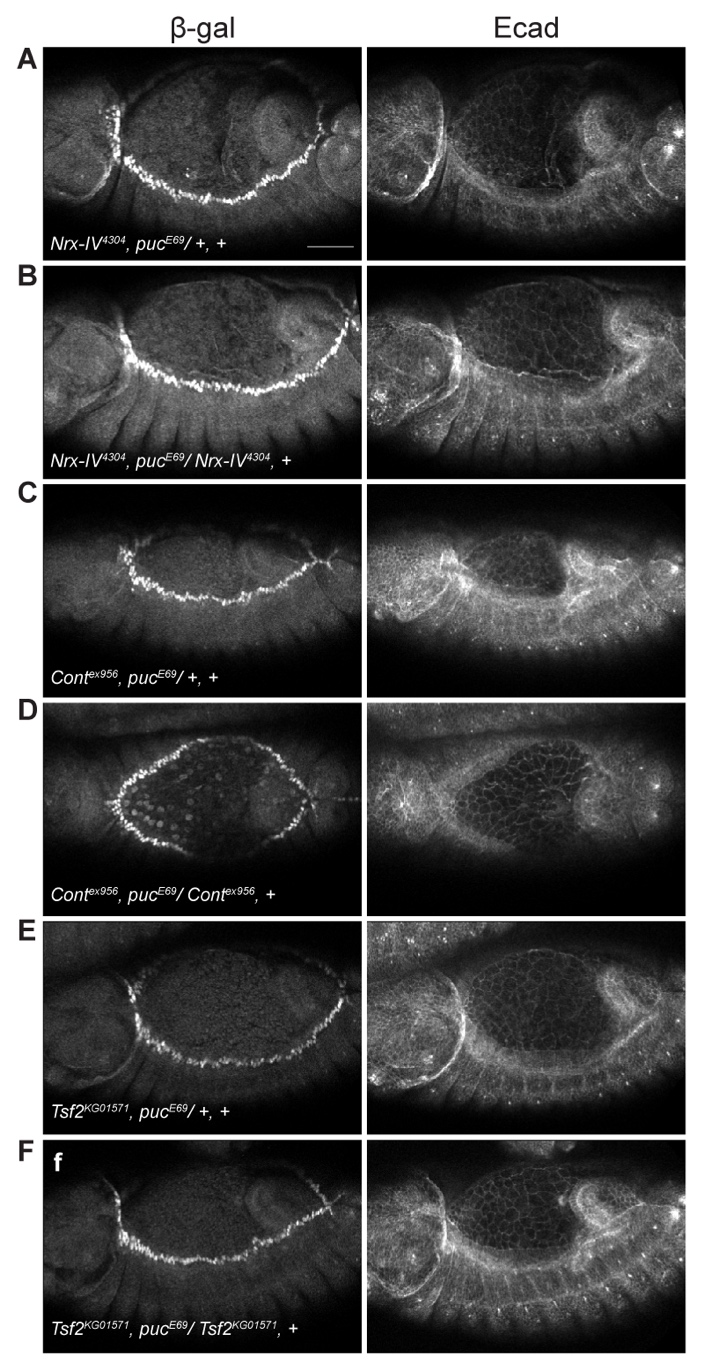
**

**Figure S2.** **JNK signaling is activated normally in SJ mutants.** (**A-F)** Confocal optical sections of stage 13 *Nrx-IV^4304^*, *Cont^ex956^*, and *Tsf2^KG01571^* heterozygous and homozygous embryos, carrying *puc^E69^* enhancer trap and stained with antibodies against β-gal to visualize *puc* expressing cells and Ecad to outline cells. (**A, C, E)** *Nrx-IV^4304^*, *Cont^ex956^*, and *Tsf2^KG01571^* heterozygous embryos exhibit a strong expression of *puc* in the dorsal-most epidermal cells. This expression is robust throughout closure. (**B, D, F)** *Nrx-IV^4304^*, *Cont^ex956^*, and *Tsf2^KG01571^* homozygous embryos also express *puc* in the dorsal-most epidermal cells and do not have any defects in expression in comparison to their respective heterozygous counterparts. Scale bar = 50 μm.


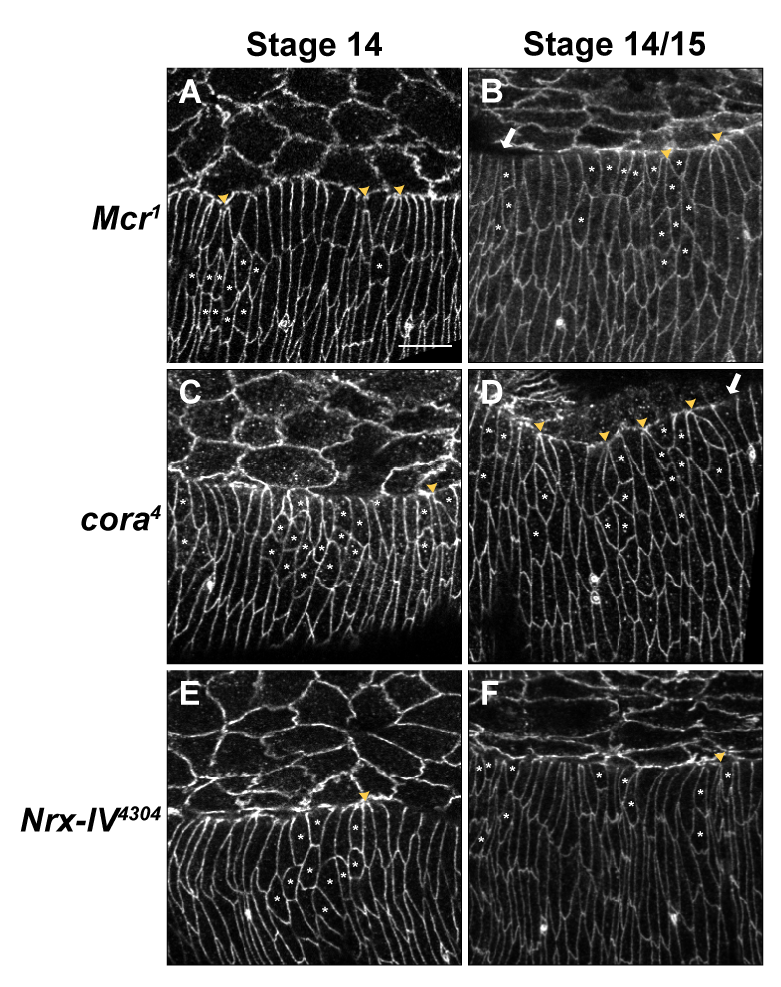


**Figure S3.** **Some SJ mutant embryos exhibit severe irregularities in the leading edge and defects in cell shape in the lateral epidermis, late in closure.** (A-F) Confocal optical sections of stage 14 and 14-15 *Mcr^1^*, *cora^4^,* and *Nrx-IV^4304^* embryos stained with antibody against E-cadherin. In some stage 14 and stage 14-15 SJ mutant embryos, many epidermal cells fail to elongate (white asterisks). Also, bunching of groups of cells at the leading edge was observed in some embryos (yellow arrowheads), along with tearing at the leading edge (white arrows). Scale bar = 10 µm.


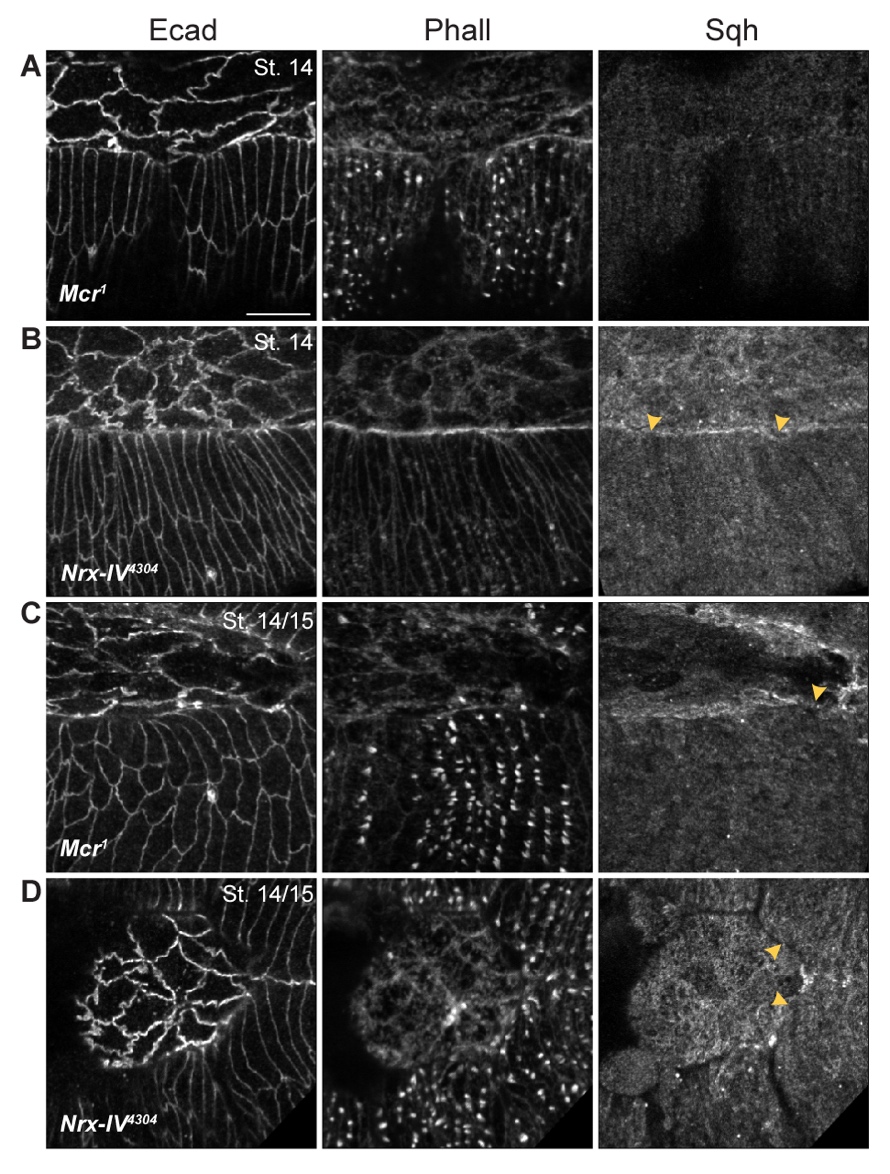


**Figure S4. *Mcr^1^* and *Nrx-IV^4304^* embryos exhibit defects in F-actin and Sqh distribution at the leading edge, late in closure.** **(A-D)** Confocal optical sections of stage 14, and 14-15 *Mcr^1^* and *Nrx-IV^4304^* embryos stained with Alexa Fluor 555 Phalloidin (Phall) and antibodies against E-cadherin and Sqh. (**A-B)** Stage 14 *Mcr^1^* and *Nrx-IV^4304^* embryos have a reduction in F-actin accumulation at the leading edge. Sqh distribution at the leading edge is diffuse with regions of discontinuity (yellow arrowheads). (**C-D)** Stage 14-15 *Mcr^1^* and *Nrx-IV^4304^* have a severe reduction in F-actin expression at the leading edge, lateral epidermis and amnioserosa. Sqh expression is also dramatically reduced with regions of complete loss (yellow arrowheads). Scale bar = 10 µm.

**
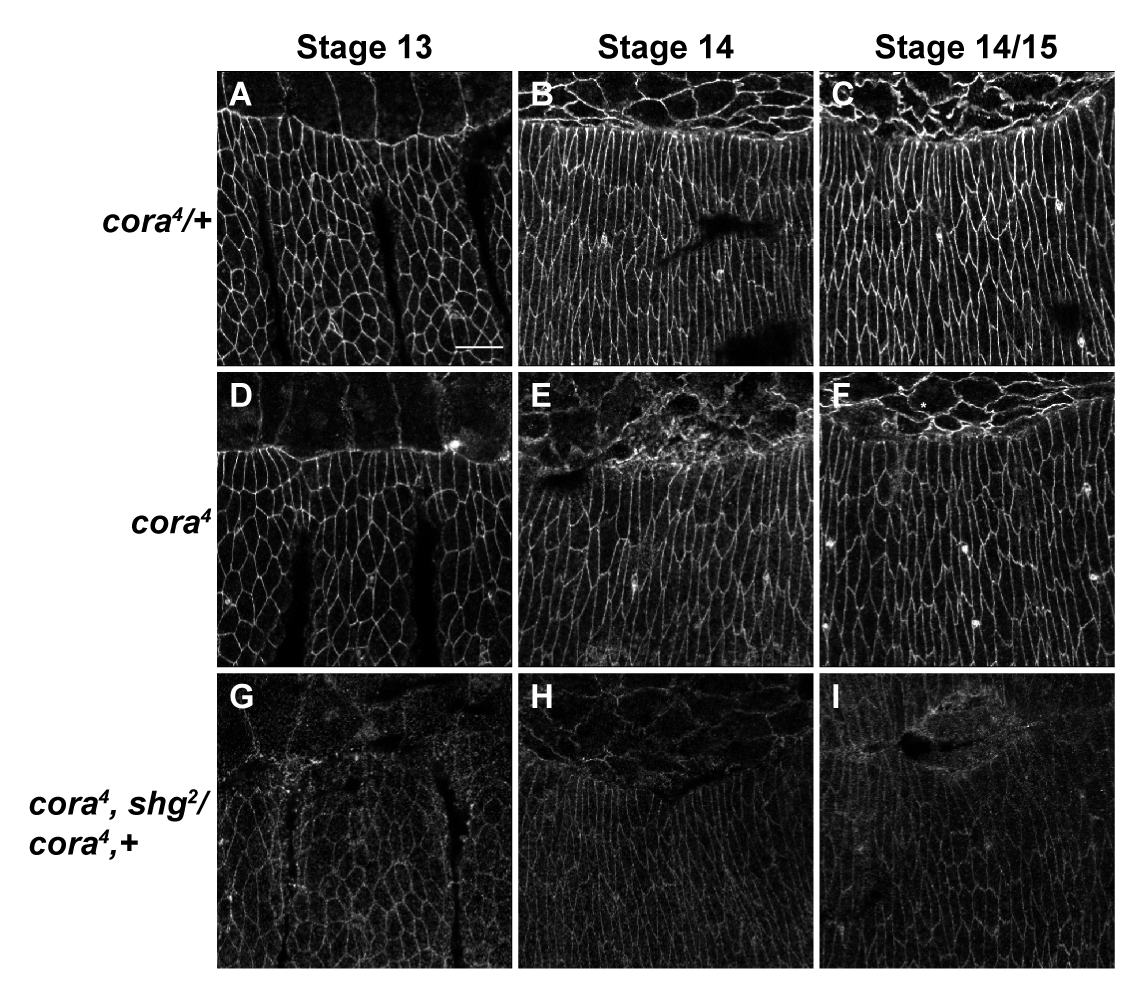
**

**Figure S5. Reducing E-cadherin in *cora^4^* embryos. (A-I)** Confocal optical sections of stage 13, stage14, and stage14-15 *cora^4^/+*, *cora^4^*, and *cora^4^*, *shg^2^/ cora^4^,+* embryos stained with an antibody against E-cadherin. **(A-C)** In wild type embryos, the intensity of E-cadherin staining seems to increase as DC progresses. **(D-F)** *cora^4^* embryos have slight reduction in E-cadherin staining in comparison to wild type embryos. **(G-I)** A heterozygous mutation in *shg* in *cora* embryos results in severe reduction in E-cadherin staining. Scale bar = 10 µm.


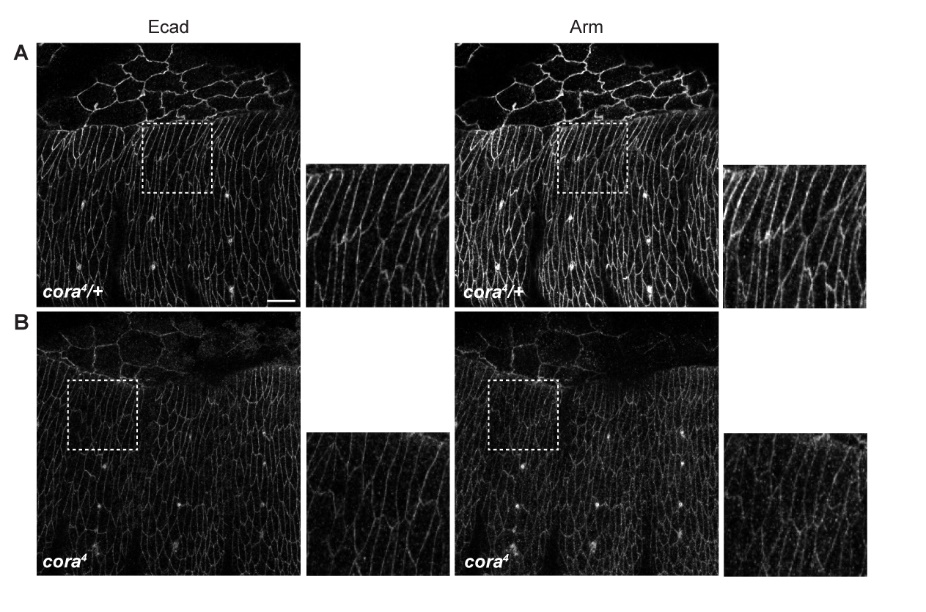


**Figure S6. Distribution of adherens junction proteins is affected in *cora^4^* mutants. (A-B)** Confocal optical sections of *cora^4^/+* (control) and *cora^4^* mutant embryos late in DC (dorsal gap length < 190μm), stained with antibodies against adherens junction components, E-cadherin and Armadillo. **(A)** In *cora^4^/+* embryos, both Ecad and Arm were tightly localized to cell junctions. **(B)** In contrast, in *cora^4^* embryos, both Ecad and Arm expression was weak at the cell junctions in comparison to controls. Also, Ecad and Arm was found to be accumulated in cytoplasmic puncta in *cora^4^* mutant epidermal cells (zoomed in section). N= 7 control and 8 *cora^4^* mutant embryos. Scale bar = 10μm.


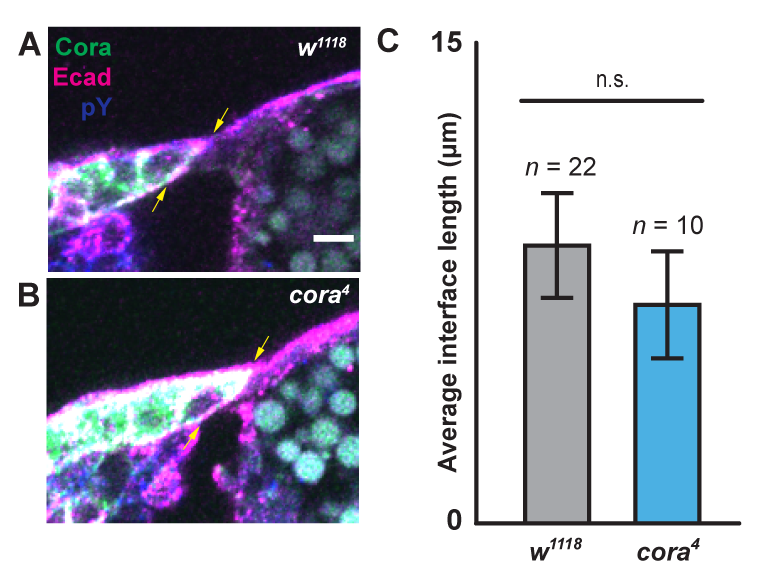


**Figure S7. Basal adhesion between the amnioserosa and lateral epidermis is unaltered in SJ mutants.** (A-B) Confocal optical images of sliced sections of wild type and *cora^4^* embryos showing the interface between the epidermis and amnioserosa, stained with antibodies against Cora (green), Ecad (red) and phosphotyrosine (pY; blue). White arrows point to length of the epidermis-amnioserosa interface. Scale bar = 5 µm. (C) Measurement of the length of the epidermis-amnioserosa interface in wild type and *cora^4^* mutant embryos. No significant difference was observed.
